# A Xanthene‐Based Mono‐Anionic PON Ligand: Exploiting a Bulky, Electronically Unsymmetrical Donor in Main Group Chemistry

**DOI:** 10.1002/chem.202004741

**Published:** 2021-01-14

**Authors:** Xiongfei Zheng, Andreas Heilmann, Caitilín McManus, Simon Aldridge

**Affiliations:** ^1^ Inorganic Chemistry Laboratory Department of Chemistry University of Oxford South Parks Road Oxford OX1 3QR UK

**Keywords:** amide ligands, gallium, low-valent compounds, oxidation, xanthene ligands

## Abstract

The synthesis of a novel mono‐anionic phosphino‐amide ligand based on a xanthene backbone is reported, togetherr with the corresponding Ga^I^ complex, (PON)Ga (PON = 4‐(di(2,4,6‐trimethylphenyl)phosphino)‐5‐(2,6‐diisopropylanilido)‐2,7‐di‐*tert*‐butyl‐9,9‐dimethylxanthene). The solid‐state structure of (PON)Ga (obtained from X‐ray crystallography) reveals very weak O⋅⋅⋅Ga and P⋅⋅⋅Ga interactions, consistent with a R_2_NGa fragment which closely resembles those found in one‐coordinate amidogallium systems. Strong N‐to‐Ga π donation from the amido substituent is reflected in a very short N−Ga distance (1.961(2) Å), while the P⋅⋅⋅Ga contact (3.076(1) Å) is well outside the sum of the respective covalent radii. While the donor properties of the PON ligand towards Ga^I^ are highly unsymmetrical, oxidation to Ga^III^ leads to much stronger coordination of the pendant phosphine as shown by P−Ga distances which are up to 20 % shorter. From a steric perspective, the PON ligand is shown to be significantly bulkier than related β‐diketiminate systems, a finding consistent with reactions of (PON)Ga towards O‐atom sources that proceed without oligomerization. Despite this, the enhanced P‐donor properties brought about by oxidation at gallium are not sufficient to quench the reactivity of the highly polar Ga−O unit. Instead, intramolecular benzylic C−H activation is observed across the Ga−O bond of a transient gallanone intermediate.

## Graphical abstract

**The application** of a sterically demanding mono‐anionic PON ligand in gallium chemistry has been probed, revealing a highly unsymmetrical donor profile towards Ga^I^ and structural features reminiscent of one‐coordinate gallium amides. Oxidation at the metal leads to a marked increase in the donor capabilities of the phosphine donor, although the transient gallanone formed by O‐atom transfer still undergoes facile C−H activation across the Ga−O bond.

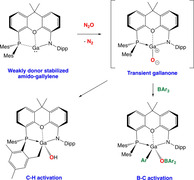

## Introduction

**Figure 1:**
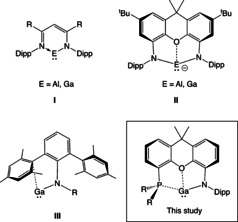
Low valent group 13 metal systems relevant to the current study.

In recent years, β‐diketiminate (′Nacnac′) ligands have been extensively used in coordination chemistry, to support a wide range of metal complexes from across the Periodic Table.[^1^, ^2^, ^3^, ^4^] Within main group chemistry, a number of landmark compounds have been reported incorporating these chelating monoanionic LX ligand systems. These include Group 13 metal complexes in the +1 oxidation state—systems which are challenging both in terms of their intrinsic tendency to undergo disproportionation and their lability towards external reagents.^[5]^ Thus in 2000, the groups of Roesky and Power successfully synthesized the monomeric Al^I^ and Ga^I^ compounds [HC{(Me)C(Dipp)N}_2_]E (or (Nacnac)E, **I**, where E=Al, Ga) using sterically encumbered Dipp‐substituted β‐diketiminate ligands (Figure 1; Dipp=2,6‐*i*Pr_2_C_6_H_3_).[^6^, ^7^, ^8^]

In 2018, we employed a chelating dianionic diamido (X_2_) ligand, [NON]^2−^, to access *anionic* Al^I^ and Ga^I^ compounds of the type K_2_[(NON)E]_2_ (**II**, E=Al, Ga; NON=4,5‐bis(2,6‐ diiso‐propyl‐anilido)‐2,7‐di‐*tert*‐butyl‐9,9‐dimethylxanthene).[^9^, ^10^, ^11^] These systems show unusual patterns of reactivity: the aluminyl anion can act as a nucleophile in the formation of C−Al and M−Al bonds,[^9^, ^12^] and effect the reversible oxidative addition of the C−C bond in benzene.^[13]^ Given the track record of the Nacnac ligand family in supporting charge neutral E(I) systems, however,[^2^, ^3^, ^4^] we targeted related mono‐anionic LX donors based on the dimethylxanthene backbone, in which one of the amido groups of the [NON]^2−^ system is formally replaced by a neutral donor. Moreover, given the successful development by Power, and by Jones, of one‐coordinate Ga^I^ amides (stabilized to a greater or lesser extent by interactions with the flanking aryl substituents, **III**),^[14]^ we targeted xanthene systems featuring L substituents which are known to act as relatively weak donors towards Ga^I^. Our aim was to develop amido complexes featuring a weakly interacting (or hemi‐labile) L donor that might display the high levels of reactivity associated with genuine one‐coordinate systems, while retaining some of the ground state stability of Nacnac compounds. Accordingly, we report in the current manuscript on the development of a mono‐anionic [PON]^−^ ligand (Figure 1) and the use of Ga^I^ and Ga^III^ chemistry to probe its coordination capabilities.

## Results and Discussion

### (i) Synthesis of H(PON) and (PON)Ga

**Figure 2:**
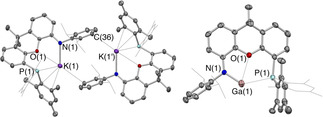
Molecular structures of K_2_(PON)_2_ (**3**; left) and (PON)Ga (**4**; right) in the solid state as determined by X‐ray crystallography. Thermal ellipsoids set at the 40 % probability level; H atoms and solvent molecules omitted and *i*Pr/selected Mes groups shown in wireframe format for clarity. Key bond lengths (Å) and angles (°): (for **3**) K(1)−N(1) 2.562(2), K(1)−O(1) 3.022(2), K(1)−P(1) 3.351(1), K(1′)−C(36) 3.308(3); (for **4**) Ga(1)−N(1) 1.961(2), Ga(1)−O(1) 2.631(2), Ga(1)−P(1) 3.076(1), N(1)‐Ga(1)‐P(1) 116.4(1).

The H(PON) protio‐ligand **2** can be synthesized in good yield (ca. 70 %) from the (known) 4‐bromo‐5‐(dimesitylphosphino)‐xanthene precursor (**1**)^[15]^ and DippNH_2_ via a Buchwald‐Hartwig coupling reaction. **2** has been characterized by multinuclear NMR, elemental microanalysis and single crystal X‐ray diffraction (see SI). Moreover, it is conveniently deprotonated by benzylpotassium, KCH_2_Ph, giving the potassiated ligand K(PON) (**3**), which can be used for onward reaction chemistry without further purification, or recrystallized from toluene for structural and spectroscopic characterization. **3** is thus obtained free of coordinating solvent as dimeric K_2_(PON)_2_, in which K(PON) monomer units are linked in head‐to‐tail fashion through close contacts between the potassium centre of one unit and the Dipp aromatic π system of the other (Figure 2).

**Scheme 1:**
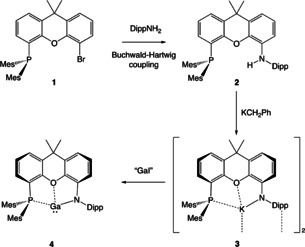
Synthesis of PON‐supported gallylene **4**.

With this new ligand system in hand we set out to probe its coordination chemistry, using gallanediyl (Ga^I^) and gallium oxide (Ga^III^) systems as probes of its ability to support highly reactive main group metal centres. Accordingly, the Ga^I^ system, (PON)Ga, **4** can be prepared in ca. 60 % isolated yield via a salt‐metathesis reaction between **3** and “GaI” (Scheme 1),^[16]^ and obtained in crystalline form by recrystallization from benzene. The ^1^H NMR spectrum of **4** features a similar pattern of resonances to those of H(PON) and K(PON), with equivalent phosphorus‐bound mesityl substituents, Dipp *i*Pr groups and xanthene backbone methyl groups implying the presence of a plane of symmetry on the NMR timescale. The ^31^P{^1^H} resonance of **4** (at δ_P_=−34.3 ppm) is very close to those of protio‐ligand **2** (δ_P_=−36.9 ppm) and potassiated system **3** (δ_P_=−34.4 ppm), implying that the interaction of the phosphine donor with the gallium centre in **4** is relatively weak. Consistently, the (monomeric) structure of **4** in the solid state (Figure 2) features a very long Ga⋅⋅⋅P distance (3.076(1) Å) which is comfortably outside the sum of the respective covalent radii (1.22(4)+1.07(3) Å),^[17]^ and much longer than that measured for the corresponding Ga^III^ system (PON)GaI_2_ which was synthesized for comparative purposes (2.612(1) Å; see SI). The Ga−O separation involving the xanthene ether linkage is also relatively long (2.631(2) Å) compared to the sum of the respective covalent radii (1.22(3)+0.66(2) Å),^[17]^ and is broadly comparable to that measured for the potassium gallyl system K_2_[(NON)Ga]_2_, featuring the related [NON]^2−^ diamide ligand (2.542(2) Å).^[9]^ On the other hand, the Ga−N bond is very short (1.961(2) Å) in comparison with those in K_2_[(NON)Ga]_2_ (2.093(2) and 2.106(2) Å),^[9]^ presumably reflecting the fact that there is only one Ga‐X covalent bond in **4**. Indeed, the Ga−N separation in **4** is in line with the bond lengths reported by Power and by Jones for Ga^I^ amides with geometries which approach mono‐coordinate (1.954(2) −1.985(4) Å),^[14]^ consistent with idea that the coordination of the phosphine and ether donors in **4** is very weak.

### (ii) Electronic and steric properties of (PON)Ga

**Figure 3:**
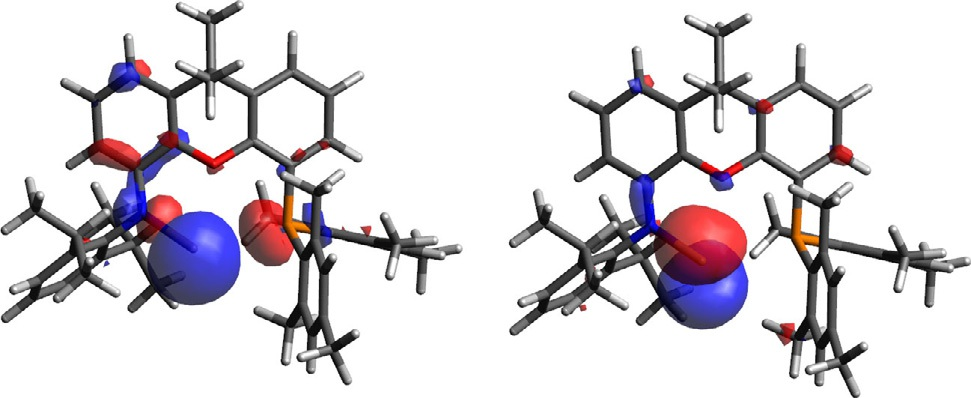
Frontier orbitals of (PON)Ga as determined by Density Functional Theory (isosurface 0.04): (left) HOMO‐1 (−5.67 eV); (right) LUMO+2 (−0.68 eV).

The electronic structure of (PON)Ga (**4**) was probed using Density Functional Theory at the PBE1PBE/TZVP level. These calculations suggest that the LUMO+2 (−0.68 eV) is comprised predominantly of gallium *p_z_* character (Figure 3). The HOMO is ligand‐based, with the orbital displaying gallium‐centred lone pair character being the HOMO‐1 (−5.67 eV). The associated energy separation (4.99 eV) is very similar to that calculated for (Nacnac)Ga^[6]^ using the same method (4.86 eV). The essentially identical energies of the formally vacant pπ orbital in each case (−0.66 eV for (Nacnac)Ga) presumably reflect the fact that in **4**, the extent of π donation from the (single) amido substituent is markedly enhanced, consistent with the very short crystallographically determined Ga−N bond length. Both of these systems have wider HOMO–LUMO gaps than that determined for the anionic diamido system [(NON)Ga]^−^. In that case, the narrow HOMO–LUMO gap (4.21 eV) reflects the fact that the LUMO is not destabilized to the same degree by N‐to‐Ga π donation due to the constraints of the xanthene backbone (which mean that the amido groups cannot attain co‐ planarity with the GaN_2_ unit).[^9^, ^11^] The (PON)Ga system can also be compared with Jones’ one‐coordinate amidogallium(I) system,^[14b]^ which is similar in that it features π stabilization from only a single N donor. In that system, the HOMO–LUMO gap (calculated using the same method) is narrower (4.62 eV) and the LUMO somewhat lower than in **4** (−0.91 eV). This presumably reflects the fact that the LUMO is orthogonal to the amido π system, and (compared to **4**) has less opportunity for interaction with ancillary neutral donors (featuring as it does, only very weak interaction with the flanking arene π system).

**Figure 4:**
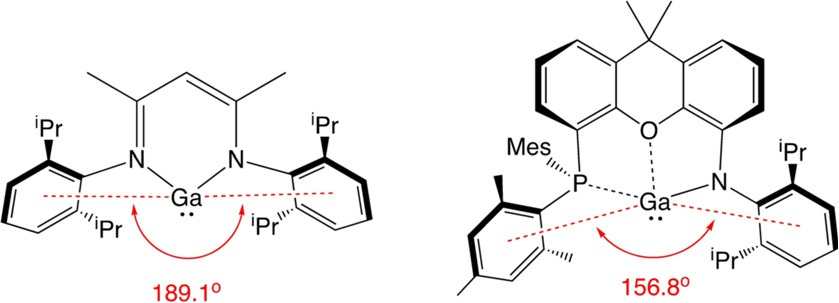
Comparison of the aryl‐flanked “pockets” adjacent to the metal centre in (Nacnac)Ga and (PON)Ga (**4**).

Steric factors are known to be a significant influence on the patterns of reactivity displayed by low‐valent main group species. In both (PON)Ga and (Nacnac)Ga, the Ga^I^ centre sits in a “pocket” between flanking aryl substituents: in the β‐diketiminate system these are the N‐bound Dipp groups, which are aligned such that the distances from the arene centroids to the metal are each ca. 4.0 Å, and the “pocket” defined by the open centroid‐Ga‐centroid angle occupies 189.1° in angular terms (Figure 4).^[6]^ In **4** the gallium centre is flanked by NDipp and PMes substituents (the centroids of which are also ca. 4.0 Å from the metal centre), and the open “pocket” by comparison occupies 156.8°. As such, we hypothesized that **4** might offer the possibility of a more sterically shielded pocket in which to carry out chemistry at the metal centre.

### (iii) Oxidation of (PON)Ga

To probe the idea of enhanced steric bulk and encouraged by the finding that the weakly‐bound phosphine donor becomes more strongly stabilizing on oxidation of the metal centre (cf. (PON)Ga and (PON)GaI_2_), we examined the reactivity of **4** towards oxygen atom transfer agents. The isolation of a (hitherto unknown) gallanone complex containing a terminal GaO moiety, would presumably require both very strongly σ‐donating and sterically demanding ancillary ligands.^[18]^

**Scheme 2:**
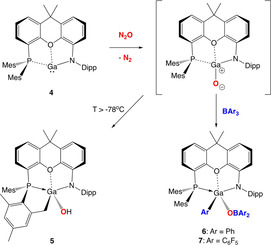
Oxidation of (PON)Ga by N_2_O: formation of products derived from intramolecular C−H or intermolecular B−C bond activation at a putative gallanone intermediate.

**Figure 5:**
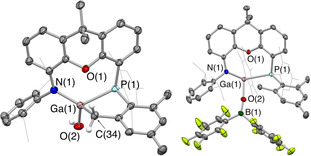
Molecular structures of C−H and B−C activated species **5** (left) and **7** (right) in the solid state as determined by X‐ray crystallography. Thermal ellipsoids set at the 40 % probability level; most H atoms and solvent molecules omitted and *i*Pr/selected Mes groups shown in wireframe format for clarity. Key bond lengths (Å) and angles (°): (for **5**) Ga(1)−N(1) 1.921(3), Ga(1)−P(1) 2.468(1), Ga(1)−O(1) 2.578(2), Ga(1)−O(2) 1.828(2), Ga(1)−C(34) 1.972(4); (for **6**) Ga(1)−N(1) 1.904(2), Ga(1)−P(1) 2.591(1), Ga(1)−O(1) 2.566(2), Ga(1)−O(2) 1.864(2), B(1)−O(2) 1.297(4), Ga(1)−C(28) 2.015(4).

Power et al. have reported the formation of [(Nacnac)Ga(μ‐O)]_2_ from the reaction of (Nacnac)Ga with N_2_O,^[19]^ with the oxo‐bridged structure presumably reflecting a steric profile which permits ready dimerization. In contrast, oxidation of **4** with N_2_O or Me_3_NO at room temperature leads to a monomeric product (**5**, Scheme 2), albeit one which results from intramolecular C−H activation of one of the *ortho*‐methyl groups of the PMes_2_ substituent across the transient gallium oxo unit.^[20]^ Compound **5** has been characterized by spectroscopic methods and its structure in the solid state confirmed crystallographically (Figure 5). The formation of **5** is signalled by the appearance of a ^31^P NMR resonance at δ_p_ = −41.0 ppm and by increased complexity in the pattern of ^1^H NMR signals associated with the *ortho* methyl substituents. Its structure in the solid state is based around an approximately tetrahedral gallium centre, featuring a significantly shortened Ga−P distance (2.468(1) Å cf. 3.076(1) Å for (PON)Ga), but with a weak Ga⋅⋅⋅O contact which is essentially unchanged from its precursor (2.578(2) Å vs. 2.631(2) Å). The Ga−C and Ga‐OH bond lengths (1.972(4) and 1.828(2) Å) fall within the ranges previously reported for related (Nacnac)Ga‐containing compounds.^[21]^

Nikonov and co‐workers have recently reported the trapping of the monomeric [(Nacnac)GaO] fragment by reactions of (Nacnac)Ga with N_2_O in the presence of a range of relatively acidic C−H bonds (e.g. those in sulfoxides and ketones, and at the 2‐position of pyridine and related heterocycles).^[20]^ In the case of **4**/**5**, the fact that C−H activation occurs in the absence of an external trap (and at an unactivated methyl group) suggests that dimerization of the putative [(PON)GaO] monomer is less facile than in the related Nacnac system—in line with estimates of the comparative sizes of the ligand‐enforced “pockets”.

Attempts to identify a labile gallanone intermediate preceding the formation of **5** by VT‐NMR methods were unsuccessful, so alternative (chemical) trapping methods were pursued via coordination of either a Lewis acid (at O) or a Lewis base (at Ga). The latter strategy led either to no change in the outcome of the reaction (using IPrMe) or to the precipitation of gallium metal in the presence of the less bulky NHC IMe_4_. On the other hand, addition of N_2_O in the presence of a borane Lewis acid (either B(C_6_F_5_)_3_ or BPh_3_) leads to the clean formation of new products which are characterized by ^31^P NMR signals at −36.1 and −35.6 ppm, respectively. Both products could be unambiguously characterized by X‐ray crystallography, and are shown to result from cleavage of a B−C bond in BPh_3_ (**6**) or B(C_6_F_5_)_3_ (**7**) across the Ga−O unit formed in the initial reaction of gallylene **4** with N_2_O (see Scheme 2, Figure 5 and SI). As such, the isolation of compounds **6** and **7** provides further evidence for the formation of a transient, highly reactive gallanone species by oxygen atom transfer to the gallium centre of **4**. It is worth noting that similar reactivity—leading to the cleavage of one of the B−C bonds in B(C_6_F_5_)_3_ across a terminal Si=O bond—has been reported by Iwamoto and co‐workers in studies of a reactive (but isolable) silanone.^[22]^

## Conclusion

In summary, we report on the development of a mono‐anionic phosphino‐amide ligand based on a xanthene backbone, togetherr with the synthesis of the corresponding Ga^I^ complex, (PON)Ga (**4**), as a probe of its coordination properties in low valent main group chemistry. Structural studies of **4** are consistent with very weak O⋅⋅⋅Ga and P⋅⋅⋅Ga interactions, and with an R_2_NGa fragment that closely resembles those found in one‐coordinate amidogallium systems. Strong N‐to‐Ga π donation from the single amido group leads to a very short N‐Ga distance (1.961(2) Å), while the P⋅⋅⋅Ga contact (3.076(1) Å) is comfortably outside the sum of the respective covalent radii. While the electronic properties of the PON donor are shown to be highly unsymmetrical towards Ga^I^, oxidation to Ga^III^ leads to stronger coordination of the pendant phosphine arm. From a steric perspective, the profile of the PON ligand is shown to be significantly larger than those of related *N*,*N′*‐chelated β‐diketiminate systems, and underpins reactivity of (PON)Ga towards O‐atom sources that proceeds without oligomerization. However, the enhanced P‐donor properties seen on oxidation are not sufficient to quench the reactivity of the highly polar Ga−O unit. Instead, intramolecular benzylic C−H activation is observed across the Ga−O bond of a transient gallanone intermediate. Further studies of the coordination chemistry of monoanionic [PON]^−^ (and related) ligands towards main group E(I) centres are in progress and will be reported in due course.

## Experimental Section

### General procedures

All manipulations were carried out using standard Schlenk line or dry‐box techniques under an atmosphere of argon or dinitrogen. Solvents were degassed by sparging with argon and dried by passing through a column of the appropriate drying agent. NMR spectra were measured in [D_6_]benzene (which was dried over potassium), with the solvent then being distilled under reduced pressure and stored under argon in Teflon valve ampoules. NMR samples were prepared under argon in 5 mm Wilmad 507‐PP tubes fitted with J. Young Teflon valves. ^1^H, ^31^P{^1^H}, ^13^C{^1^H}, ^11^B{^1^H}, ^19^F{^1^H} NMR spectra were measured on a Bruker Avance III HD nanobay 400 MHz or Bruker Avance 500 MHz spectrometer at ambient temperature and referenced internally to residual protio‐solvent (^1^H) or solvent (^13^C) resonances and are reported relative to tetramethylsilane (*δ*=0 ppm). Assignments were confirmed using two‐dimensional ^1^H‐^1^H and ^13^C‐^1^H NMR correlation experiments. Chemical shifts are quoted in δ (ppm) and coupling constants in Hz. Elemental analyses were carried out by Elemental Microanalysis Ltd, Okehampton, Devon, UK. Compound **1**,^[15]^ B(C_6_F_5_)_3_ ^[23]^ and KCH_2_Ph^[24]^ were prepared by literature methods. All other reagents were used as received. The synthetic and characterizing data for H(PON) (**2**) and (PON)GaI_2_ are included in the Supporting Information.

### Crystallographic details

Single‐crystal X‐ray diffraction data for compounds **2**–**7** and (PON)GaI_2_ were collected at 150 K on an Oxford Diffraction/Agilent SuperNova diffractometer using Cu‐*Kα* radiation (*λ*=1.54184 Å), and equipped with a nitrogen gas Oxford Cryosystems cooling unit.^[25]^ Raw frame data were reduced using CrysAlisPro.^[26]^ The structures were solved using SHELXT^[27]^ and refined to convergence on *F* ^2^ by full‐matrix least‐squares using SHELXL^[28]^ in combination with OLEX2.^[29]^ Distances and angles were calculated using the full covariance matrix. Restraints were used to maintain sensible geometries for the disordered groups and approximate the displacement parameters to typical values. Selected crystallographic data are summarized in the Table S1. Deposition Numbers 2036919, 2036920, 2036921, 2036922, 2036923, 2036924, and 2036925 contain the supplementary crystallographic data for this paper. These data are provided free of charge by the joint Cambridge Crystallographic Data Centre and Fachinformationszentrum Karlsruhe Access Structures service www.ccdc.cam.ac.uk/structures.

### Density functional theory (DFT) calculations

The computational work was performed using DFT within the Gaussian09 (Revision D.01) program package.^[30]^ Geometry optimizations of the monoanionic ligand systems were performed with the PBE1PBE hybrid exchange‐correlation functional^[31]^ using a TZVP basis set.^[32]^ Grimme's empirical dispersion correction (DFT‐D3) was included in all geometry optimizations.^[33]^ Unless otherwise stated, geometry optimizations were carried out for the full system, and frequency calculations were performed to confirm the nature of the stationary points found (minimum). Graphics were created with the Avogadro program.^[34]^ The natural bond orbital (NBO) analyses were performed using NBO 3.1 as implemented in Gaussian09.^[35]^

### Syntheses of novel compounds

**[K(PON)]_2_, 3**: Toluene (30 mL) was added to a Schlenk flask containing a mixture of **2** (1.01 g, 1.50 mmol) and benzylpotassium (0.21 g, 1.60 mmol). The reaction mixture was stirred for 3 h at room temperature, filtered and volatiles removed from the filtrate in vacuo to give **3** as an off‐white solid (1.02 g, 95 %). Single crystals suitable for X‐ray crystallography were obtained by slow evaporation from a toluene solution. ^1^H NMR (400 MHz, C_6_D_6_, 298 K): *δ*=1.05 (d, *J*_HH_=6.9 Hz, 6 H, CH(C*H*_3_)_2_), 1.27 (d, *J*_HH_=6.9 Hz, 6 H, CH(C*H*_3_)_2_), 1.73 (s, 6 H, C*H*_3_ of XA), 2.00 (s, 12 H, *o*‐C*H*_3_ of Mes), 2.09 (s, 6 H, *p*‐C*H*_3_ of Mes), 3.26 (sept, *J*_HH_=6.9 Hz, 2 H, C*H*(CH_3_)_2_), 6.17 (dd, *J*_HH_=1.2 Hz, *J*_HH_=8.0 Hz, 1 H, Ar*H*), 6.20 (dd, *J*_HH_=1.2, 8.0 Hz, 1 H, Ar*H*), 6.66 (d, *J*_HH_=2.8 Hz, 4 H, C*H* of Mes), 6.72 (t, *J*_HH_=7.8 Hz, 1 H, Ar*H*), 7.05 (m, 2 H, Ar*H*), 7.19 (d, *J*_HH_=7.6 Hz, 1 H, Ar*H*), 7.30 (d, *J*_HH_=7.7 Hz, 1 H, Ar*H*), 7.35 (d, 7.6 Hz, 2 H, Ar*H*) ppm. ^13^C NMR (126 MHz, C_6_D_6_, 298 K): *δ*=156.6 (d, *J*_PC_=18.6 Hz), 153.6, 149.5, 143.9, 142.7 (d, *J*_PC_=14.7 Hz), 139.2, 138.5, 137.9 (toluene), 133.5 (d, *J*_PC_=2.1 Hz), 132.0, 131.0 (d, *J*_PC_=3.8 Hz), 130.5 (d, *J*_PC_ 3.7 Hz), 130.2 (d, J_PC_=10.3 Hz), 129.3 (toluene), 128.7, 128.6 (toluene), 125.7 (toluene), 125.3, 123.7, 122.4, 120.8 (d, J_PC_=6.4 Hz), 120.0, 110.6, 101.2, 34.8 (d, *J*_PC_=2.0 Hz), 32.7, 27.9, 25.2, 24.9, 22.9, 22.8, 21.4 (toluene), 20.9 ppm. ^31^P NMR (162 MHz, C_6_D_6_, 298 K): *δ*=−34.4 ppm.

**(PON)Ga, 4**: To a mixture of Ga metal (0.12 g, 1.70 mmol) and iodine (0.22 g, 0.65 mmol) in a Schlenk flask was added toluene (30 mL). The mixture was sonicated until a green precipitate appeared, and a solution of **3** (1.00 g, 1.50 mmol) in toluene (20 mL) added dropwise. After stirring at room temperature overnight, the reaction mixture was allowed to settle and filtered by cannula. The resulting filtrate was concentrated (to ca. 20 mL) and crystalline product obtained by slow evaporation at room temperature. (0.67 g, 60 %). ^1^H NMR (400 MHz, C_6_D_6_, 298 K): *δ*=1.12 (d, *J*_HH_=7.1 Hz, 6 H, CH(C*H*_3_)_2_), 1.14 (d, *J*_HH_=7.0 Hz, 6 H, CH(C*H*_3_)_2_), 1.50 (s, 6 H, C*H*_3_ of XA), 2.06 (s, 6 H, *p*‐C*H*_3_ of Mes), 2.19 (s, 12 H, *o*‐C*H*_3_ of Mes), 3.40 (sept, *J*_HH_=6.8 Hz, 2 H, C*H*(CH_3_)_2_), 6.13 (dd, *J*_HH_=1.4, 8.0 Hz, 1 H, Ar*H*), 6.55 (dd, *J*_HH_=1.4, 8.0 Hz, 1 H, Ar*H*), 6.70 (d, *J*_HH_=2.8 Hz, 4 H, C*H* of Mes), 6.76 (m, 1 H, Ar*H*), 6.89 (t, *J*_HH_=7.8 Hz, 1 H, Ar*H*), 7.30 (m, 3 H, Ar*H*), 7.36 (m, 2 H, Ar*H*) ppm. ^13^C NMR (126 MHz, C_6_D_6_, 298 K): *δ*=157.4 (d, *J*_PC_=22.2 Hz), 148.9, 142.7 (d, *J*_PC_=13.5 Hz), 142.6, 142.4, 138.8, 137.3, 134.1 (d, *J*_PC_=2.8 Hz), 133.8, 131.2, 130.9 (d, *J*_PC_=4.3 Hz), 130.1 (d, *J*_PC_=9.4 Hz), 127.4, 126.4, 125.2, 124.5, 124.0, 122.0 (d, *J*_PC_=8.2 Hz), 112.8, 109.9, 35.1, 30.2, 28.4, 26.5 24.9, 23.2 (d, *J*_PC_=12.5 Hz), 21.0 ppm. ^31^P NMR (162 MHz, C_6_D_6_, 298 K): *δ*=−34.3 ppm. Elemental microanalysis: calc. C 74.80 % H 7.11 % N 1.94 %, meas. C 74.47 % H 7.06 N 1.80 %.

**Reaction of 4 with N_2_O; synthesis of C−H activation product 5**: A solution of **4** (96 mg, 0.13 mmol) in toluene (5 mL) was subjected to three freeze‐pump‐thaw cycles before backfilling with N_2_O (ca. 1 atm) at −78 °C. The solution was stirred at this temperature for 4 h, and slowly warmed up to room temperature. The resulting solutionn was kept at −30 °C for several days to afford colourless crystals of **5** (40 mg, 41 %). ^1^H NMR (400 MHz, C_6_D_6_, 298 K): *δ*=−0.11 (d, *J*_PH_=4.1 Hz, 1 H, [Ga]O*H*), 0.97 (d, *J*_HH_=6.6 Hz, 3 H, CH(C*H*_3_)_2_), 1.03 (d, *J*_HH_=6.6 Hz, 3 H, CH(C*H*_3_)_2_), 1.08 (s, 3 H, C*H*_3_ of XA), 1.28 (d, *J*_HH_=7.0 Hz, 3 H, CH(C*H*_3_)_2_), 1.42 (s, 3 H, C*H*_3_ of XA), 1.53 (d, *J*_HH_=7.0 Hz, 3 H, CH(C*H*_3_)_2_), 1.97 (s, 3 H, *o*‐C*H*_3_ of Mes), 2.01 (m, 6 H, *o*‐C*H*_3_ of Mes), 2.16 (s, 3 H, *p*‐C*H*_3_ of Mes), 2.25 (s, 3 H, *p*‐C*H*_3_ of Mes), 2.82 (m, 2 H, [Ga]C*H*_2_), 3.48 (sept, *J*_HH_=6.9 Hz, 1 H, C*H*(CH_3_)_2_), 3.88 (sept, *J*_HH_=6.9 Hz, 1 H, C*H*(CH_3_)_2_), 6.30 (dd, *J*_HH_=1.3, 8.2 Hz, 1 H, Ar*H*), 6.45 (s, 1 H, Ar*H*), 6.50 (dd, *J*_HH_=1.3, 7.8 Hz, 1 H, Ar*H*), 6.56 (m, 1 H, Ar*H*), 6.64 (m, 1 H, Ar*H*), 6.82 (td, *J*_HH_=1.2, 7.6 Hz, 1 H, Ar*H*), 6.89 (t, *J*_HH_=8.0 Hz, 1 H, Ar*H*), 7.13 (m, 3 H, Ar*H*), 7.22 (t, *J*_HH_=7.6 Hz, 1 H, Ar*H*), 7.30 (m, 1 H, Ar*H*), 7.45 (td, *J*_HH_=1.3, 7.8 Hz, 1 H, Ar*H*) ppm. ^13^C NMR (125.8 MHz, C_6_D_6_, 298 K): *δ*=155.3 (d, *J*_PC_=13.5 Hz), 151.3, 151.1, 148.9, 147.8, 143.8, 143.7 (d, *J*_PC_=8.7 Hz), 143.1, 141.8, 141.2, 141.0, 140.9, 139.7, 136.5 (d, *J*_PC_=3.8 Hz), 133.0, 132.6 (d, *J*_PC_=6.3 Hz), 131.5 (d, *J*_PC_=7.2 Hz), 131.0 (d, *J*_PC_=3.6 Hz), 130.9 (d, *J*_PC_=7.5 Hz), 128.9 (d, *J*_PC_=6.0 Hz), 127.3, 126.7 (t, *J*_PC_=22.0 Hz), 125.2, 124.7 (d, *J*_PC_=16.8 Hz), 123.4 (d, *J*_PC_=5.7 Hz), 121.7 (d, *J*_PC_=31.0 Hz), 119.6, 119.3, 114.1, 109.3, 36.5, 32.0 (Hexane), 31.6, 28.2 (d, *J*_PC_=8.5 Hz), 25.3, 25.2, 25.1, 25.0, 24.5, 24.0 (d, *J*_PC_=5.7 Hz), 23.5, 23.2, 23.1 (Hexane), 21.0, 20.8, 14.4 (Hexane) ppm. ^31^P NMR (162 MHz, C_6_D_6_, 298 K): *δ*=−41.0 ppm. Elemental microanalysis: calc. C 73.18 % H 6.96 % N 1.90 %, meas. C 73.10 % H 6.67 % N 1.86 %.

**Reaction of 4 with N_2_O and BPh_3_/B(C_6_F_5_)_3_; synthesis of B‐C activation products 6 and 7**: These two reactions were carried out in a similar manner, exemplified here for compound **6**. A mixture of **4** (71 mg, 0.098 mmol) and BPh_3_ (24 mg, 0.098 mmol) was dissolved in toluene (5 mL) and the resulting solution subjected to three freeze‐pump‐thaw cycles before backfilling with N_2_O (to ca. 1 atm) at −78 °C. The solution was stirred at this temperature for 4 h, and slowly warmed to room temperature. The resulting solution was kept at −30 °C for several days to afford colourless crystals of **6** (31 mg, 32 %). ^1^H NMR (400 MHz, C_6_D_6_, 298 K): *δ*=1.04 (d, *J*_HH_=6.4 Hz, 3 H, CH(C*H*_3_)_2_), 1.13 (m, 6 H, CH(C*H*_3_)_2_), 1.47 (m, 12 H, *o*‐C*H*_3_ of Mes), 1.81 (s, 3 H, C*H*_3_ of XA), 1.95 (s, 3 H, C*H*_3_ of XA), 2.09 (m, 3 H, CH(C*H*_3_)_2_), 2.37 (s, 3 H, *p*‐C*H*_3_ of Mes), 3.04 (s, 3 H, *p*‐C*H*_3_ of Mes), 3.58 (sept, *J*_HH_=6.8 Hz, 1 H, C*H*(CH_3_)_2_), 4.16 (sept, *J*_HH_=6.8 Hz, 1 H, C*H*(CH_3_)_2_), 5.98 (s, 1 H, Ar*H*), 6.37 (m, 2 H, Ar*H*), 6.55 (m, 1 H, Ar*H*), 6.67 (m, 4 H, *m‐H* of Mes), 6.84 (m, 3 H, Ar*H* of Dipp), 7.14 (m, 5 H, Ar*H* of [Ga]Ph), 7.22 (m, 5 H, Ar*H* of [Ga]OBPh), 7.28 (m, 1 H, Ar*H*), 7.37 (m, 5 H, Ar*H* of [Ga]OBPh) ppm. ^13^C NMR (125.8 MHz, C_6_D_6_, 298 K): *δ*=155.8 (d, *J*_PC_=12.1 Hz), 149.4, 148.7, 144.4, 143.8, 143.4, 142.9 (d, *J*_PC_=22.1 Hz), 142.5, 142.2, 141.7, 140.5, 140.2, 139.9, 139.3, 136.3, 135.2, 133.8, 131.9, 131.8, 131.5, 130.8, 130.4, 128.8, 128.7, 128.6 (d, *J*_PC_=4.2 Hz), 127.2, 126.4, 125.8, 125.0, 124.5, 119.4, 119.2, 116.1, 109.3, 35.7, 33.3, 32.0 (hexane), 28.7, 28.1, 26.5, 25.8, 25.6 (d, *J*_PC_=11.6 Hz), 24.1, 23.7, 23.4, 23.1 (hexane), 20.9, 20.5, 14.4 (hexane) ppm. ^31^P NMR (162 MHz, C_6_D_6_, 298 K): *δ*=−36.0 ppm. **7** was obtained from **4** (69 mg, 0.096 mmol) and B(C_6_F_5_)_3_ (49 mg, 0.096 mmol) in similar fashion as colourless crystals (41 mg, 34 %). Characterizing data for **7**: ^1^H NMR (400 MHz, C_6_D_6_, 298 K): *δ*=0.81 (d, *J*_HH_=6.8 Hz, 3 H, CH(C*H*_3_)_2_), 0.96 (d, *J*_HH_=6.6 Hz, 3 H, CH(C*H*_3_)_2_), 1.07 (d, *J*_HH_=6.6 Hz, 3 H, CH(C*H*_3_)_2_), 1.36 (s, 3 H, C*H*_3_ of Mes), 1.42 (s, 3 H, C*H*_3_ of XA), 1.45 (s, 3 H, C*H*_3_ of XA), 1.54 (d, *J*_HH_=6.8 Hz, 3 H, CH(C*H*_3_)_2_), 1.70 (s, 3 H, C*H*_3_ of Mes), 1.74 (s, 3 H, C*H*_3_ of Mes), 1.91 (s, 3 H, C*H*_3_ of Mes), 2.08 (s, 3 H, C*H*_3_ of Mes), 2.70 (s, 3 H, C*H*_3_ of Mes), 3.42 (sept, *J*_HH_=6.8 Hz, 1 H, C*H*(CH_3_)_2_), 3.64 (sept, *J*_HH_=6.8 Hz, 1 H, C*H*(CH_3_)_2_, 6.04 (m, 2 H, Ar*H*), 6.53 (m, 2 H, Ar*H*), 6.55 (dd, *J*_HH_=1.3, 7.9 Hz, 1 H, Ar*H*), 6.58 (td, *J*_HH_=1.2, 7.7 Hz, 1 H, Ar*H*), 6.68 (t, *J*_HH_=7.7 Hz, 1 H, Ar*H*), 6.81 (m, 3 H, Ar*H*), 6.88 (dd, *J*_HH_=1.7, 7.7 Hz, 1 H, Ar*H*), 7.05 (d, *J*_HH_=7.7 Hz, 1 H, Ar*H*), 7.12 (t, *J*_HH_=7.7 Hz, 1 H, Ar*H*) ppm. ^13^C NMR (126 MHz, C_6_D_6_, 298 K): *δ*=154.8 (d, *J*_PC_=9.8 Hz), 148.7, 148.4, 147.7, 145.8, 143.4 (d, *J*_PC_=14.6 Hz), 142.6, 141.9, 141.8, 141.6 (d, *J*_PC_=2.4 Hz), 141.2, 141.0, 140.7, 139.6 (d, *J*_PC_=4.1 Hz), 139.3, 138.3, 136.3, 135.0 (d, *J*_PC_=3.5 Hz), 132.7, 131.5 (d, *J*_PC_=6.8 Hz), 131.2 (d, *J*_PC_=2.8 Hz), 131.2, 131.1 (d, *J*_PC_=2.3 Hz), 129.2, 126.8, 125.4, 125.1, 124.8, 124.7 (d, *J*_PC_=6.2 Hz), 124.1, 122.9 (d, *J*_PC_=33.5 Hz), 116.7, 116.4, 110.8, 35.4, 33.3, 32.0 (Hexane), 28.1, 27.5, 26.8, 25.2, 24.2, 23.9 (d, *J*_PC_=2.8 Hz), 23.9, 23.7, 23.3, 23.1, 23.0 (hexane), 22.5 (d, *J*_PC_=2.8 Hz), 20.7, 20.4, 14.4 (hexane) ppm. ^31^P NMR (162 MHz, C_6_D_6_, 298 K): *δ*=−35.6 ppm. ^19^F NMR (471 MHz, C_6_D_6_, 298 K): *δ*=−111.8 (s, 1F, *o*‐F of [Ga]C_6_F_5_), −119.3 (d, *J*_FF_=25.6 Hz, 1F, *o*‐F of [Ga]C_6_F_5_), −130.9 (s, 4F, *o*‐F of [Ga]OB(C_6_F_5_)_2_), −154.5 (t, *J*_FF_=19.5 Hz, 1F, *p*‐F of [Ga]C_6_F_5_), 154.7 (t, *J*_FF_=20.5 Hz, 2F, *p*‐F of [Ga]OB(C_6_F_5_)_2_), −161.7 (s, 1F, *m*‐F of [Ga]C_6_F_5_), −162.8 (m, 4F, *m*‐F of [Ga]OB(C_6_F_5_)_2_), −163.7 (s, 1F, *m*‐F of [Ga]C_6_F_5)_  ppm. Elemental microanalysis: calc. C 60.51 % H 4.11 % N 1.12 %, meas. C 60.83 % H 3.92 % N 1.26 %.

## Conflict of interest

The authors declare no conflict of interest.

## Supporting information

As a service to our authors and readers, this journal provides supporting information supplied by the authors. Such materials are peer reviewed and may be re‐organized for online delivery, but are not copy‐edited or typeset. Technical support issues arising from supporting information (other than missing files) should be addressed to the authors.

Supplementary

## References

[1] For early references, see:

[1a] W. Bradley , I. Wright , J. Chem. Soc. 1956, 640–648;

[1b] J. E. Parks , R. H. Holm , Inorg. Chem. 1968, 7, 1408–1416;

[1c] S. G. McGeachin , Can. J. Chem. 1968, 46, 1903–1912;

[1d] C. L. Honeybourne , G. A. Webb , Chem. Commun. (London) 1968, 739–740;

[1e] R. Bonnett , D. C. Bradley , K. J. Fisher , Chem. Commun. (London) 1968, 886–887.

[2] L. Bourget-Merle , M. F. Lappert , J. R. Severn , Chem. Rev. 2002, 102, 3031–3066. 12222981 10.1021/cr010424r

[3] For high profile examples from across the Periodic Table see, for example:

[3a] M. S. Hill , P. B. Hitchcock , R. Pongtavornpinyo , Science 2006, 311, 1904–1907; 16574862 10.1126/science.1123945

[3b] S. P. Green , C. Jones , A. Stasch , Science 2007, 318, 1754–1757; 17991827 10.1126/science.1150856

[3c] M. M. Rodriguez , E. Bill , W. W. Brenessel , P. L. Holland , Science 2011, 334, 780–783. 22076372 10.1126/science.1211906 PMC3218428

[4] For selected recent reviews encompassing aspects of Nacnac chemistry, see:

[4a] M. Asay , C. Jones , M. Driess , Chem. Rev. 2011, 111, 354–396; 21133370 10.1021/cr100216y

[4b] Y. Tsai , Coord. Chem. Rev. 2012, 256, 722–758;

[4c] C. Chen , S. M. Bellows , P. L. Holland , Dalton Trans. 2015, 44, 16654–16670; 26244489 10.1039/c5dt02215k PMC4581995

[4d] C. Camp , J. Arnold , Dalton Trans. 2016, 45, 14462–14498. 27353604 10.1039/c6dt02013e

[5] C. Dohmeier , D. Loos , H. Schnöckel , Angew. Chem. Int. Ed. Engl. 1996, 35, 129–149;

[6] N. J. Hardman , B. E. Eichler , P. P. Power , Chem. Commun. 2000, 1991–1992.

[7] C. Cui , H. Roesky , H.-G. Schmidt , M. Noltemeyer , H. Hao , F. Cimpoesu , Angew. Chem. Int. Ed. 2000, 39, 4274–4276; 10.1002/1521-3773(20001201)39:23<4274::AID-ANIE4274>3.0.CO;2-K 29711904

[8] For other examples of N-heterocyclic Ga^I^ systems relevant to this study, see ref. [4 a] and

[8a] E. S. Schmidt , A. Jockisch , H. Schmidbaur , J. Am. Chem. Soc. 1999, 121, 9758–9759;

[8b] R. J. Baker , R. D. Farley , C. Jones , M. Kloth , D. Murphy , Dalton Trans. 2002, 3844–3850;

[8c] C. Jones , P. C. Junk , J. A. Platts , A. Stasch , J. Am. Chem. Soc. 2006, 128, 2206–2207; 16478162 10.1021/ja057967t

[8d] I. L. Fedushkin , A. N. Lukoyanov , G. K. Fukin , S. Y. Ketkov , M. Hummert , H. Schumann , Chem. Eur. J. 2008, 14, 8465–8468; 18698564 10.1002/chem.200801267

[8e] I. L. Fedushkin , A. N. Lukoyanov , A. N. Tishkina , G. K. Fukin , K. A. Lyssenko , M. Hummert , H. Schumann , Chem. Eur. J. 2010, 16, 7563–7571; 20486109 10.1002/chem.201000377

[8f] Y. Liu , S. Li , X.-J. Yang , Q.-S. Li , Y. Xie , H. F. Schaefer , B. Wu , J. Organomet. Chem. 2011, 696, 1450–1455;

[8g] S. L. Choong , W. D. Woodul , A. Stasch , C. Schenk , C. Jones , Aus. J. Chem. 2011, 64, 1173–1176;

[8h] D. Dange , S. L. Choong , C. Schenk , A. Stasch , C. Jones , Dalton Trans. 2012, 41, 9304–9315; 22539449 10.1039/c2dt30299c

[8i] A. L. Hawley , C. A. Ohlin , L. Fohlmeister , A. Stasch , Chem. Eur. J. 2017, 23, 447–455. 27813169 10.1002/chem.201604495

[9] J. Hicks , P. Vasko , J. M. Goicoechea , S. Aldridge , Nature 2018, 557, 92–95. 29662211 10.1038/s41586-018-0037-y

[10] For other examples of aluminyl compounds see:

[10a] R. J. Schwamm , M. D. Anker , M. Lein , M. P. Coles , Angew. Chem. Int. Ed. 2019, 58, 1489–1493; 10.1002/anie.201811675 30548141

[10b] S. Kurumada , S. Takamori , M. Yamashita , Nat. Chem. 2020, 12, 36–39; 31767993 10.1038/s41557-019-0365-z

[10c] R. J. Schwamm , M. P. Coles , M. S. Hill , M. F. Mahon , C. L. McMullin , N. A. Rajabi , A. S. S. Wilson , Angew. Chem. Int. Ed. 2020, 59, 3928–3932; 10.1002/anie.201914986 PMC7159655 31830364

[10d] K. Koshino , R. Kinjo , J. Am. Chem. Soc. 2020, 142, 9057–9062; 32321239 10.1021/jacs.0c03179

[10e] S. Grams , J. Eyselein , J. Langer , J. Färber , S. Harder , Angew. Chem. Int. Ed. 2020, 59, 15982–15986; 10.1002/anie.202006693 PMC7540686 32449816

[11] J. Hicks , P. Vasko , J. M. Goicoechea , S. Aldridge , Angew. Chem. Int. Ed. 2020, 10.1002/anie.202007530; PMC7693242 32722863

[12] J. Hicks , A. Mansikkamäki , P. Vasko , J. M. Goicoechea , S. Aldridge , Nat. Chem. 2019, 11, 237–241. 30664716 10.1038/s41557-018-0198-1

[13] J. Hicks , P. Vasko , J. M. Goicoechea , S. Aldridge , J. Am. Chem. Soc. 2019, 141, 11000–11003. 31251586 10.1021/jacs.9b05925

[14a] R. J. Wright , M. Brynda , J. C. Fettinger , A. R. Betzer , P. P. Power , J. Am. Chem. Soc. 2006, 128, 12498–12509; 16984201 10.1021/ja063072k

[14b] D. Dange , J. Li , C. Schenk , H. Schnöckel , C. Jones , Inorg. Chem. 2012, 51, 13050–13059. 23157174 10.1021/ic3022613

[15] Z. Mo , E. L. Kolychev , A. Rit , J. Campos , H. Niu , S. Aldridge , J. Am. Chem. Soc. 2015, 137, 12227–12230. 26356306 10.1021/jacs.5b08614

[16] M. L. H. Green , P. Mountford , G. J. Smout , S. R. Speel , Polyhedron 1990, 9, 2763–2765.

[17] B. Cordero , V. Gómez , A. E. Platero-Prats , M. Revés , J. Echeverría , E. Cremades , F. Barragán , S. Alvarez , Dalton Trans. 2008, 2832–2838. 18478144 10.1039/b801115j

[18] For related aluminium oxide species, see:

[18a] J. Hicks , A. Heilmann , P. Vasko , J. M. Goicoechea , S. Aldridge , Angew. Chem. Int. Ed. 2019, 58, 17265–17268; 10.1002/anie.201910509 31550066

[18b] M. Anker , M. P. Coles , Angew. Chem. Int. Ed. 2019, 58, 18261–18265; 10.1002/anie.201911550 31568609

[19] N. J. Hardman , P. P. Power , Inorg. Chem. 2001, 40, 2474–2475. 11350220 10.1021/ic015506c

[20] A. Kassymbek , S. F. Vyboishchikov , B. M. Gabidullin , D. Spasyuk , M. Pilkington , G. I. Nikonov , Angew. Chem. Int. Ed. 2019, 58, 18102–18107; 10.1002/anie.201913028 31643119

[21a] F. Hennersdorf , J. Frötschel , J. J. Weigand , J. Am. Chem. Soc. 2017, 139, 14592–14604; 28885837 10.1021/jacs.7b07704

[21b] E. Bernabé-Pablo , V. Jancik , M. Moya-Cabrera , Inorg. Chem. 2013, 52, 6944–6950; 23724966 10.1021/ic4010054

[21c] D. Solis-Ibarra , M. d. J. Velásquez-Hernández , R. Huerta-Lavorie , V. Jancik , Inorg. Chem. 2011, 50, 8907–8917; 21851088 10.1021/ic200976d

[21d] V. Jancik , L. W. Pineda , A. C. Stückl , H. W. Roesky , R. Herbst-Irmer , Organometallics 2005, 24, 1511–1515.

[22] R. Kobayashi , S. Ishida , T. Iwamoto , Angew. Chem. Int. Ed. 2019, 58, 9425–9428; 10.1002/anie.201905198 31095845

[23] A. G. Massey , A. J. Park , J. Organomet. Chem. 1964, 2, 245–250.

[24] P. J. Bailey , R. A. Coxall , C. M. Dick , S. Fabre , L. C. Henderson , C. Herber , S. T. Liddle , D. Loroño-González , A. Parkin , S. Parsons , Chem. Eur. J. 2003, 9, 4820–4828. 14566890 10.1002/chem.200305053

[25] J. Cosier , A. M. Glazer , J. Appl. Crystallogr. 1986, 19, 105–107.

[26] CrysAlisPRO, Oxford Diffraction/Agilent Technologies UK Ltd., Yarnton, UK.

[27] G. Sheldrick , Acta Crystallogr. Sect. C 2015, 71, 3–8. 10.1107/S2053273314026370 PMC4283466 25537383

[28] G. Sheldrick , Acta Crystallogr. Sect. A 2008, 64, 112–122. 18156677 10.1107/S0108767307043930

[29] O. V. Dolomanov , L. J. Bourhis , R. J. Gildea , J. A. K. Howard , H. Puschmann , J. Appl. Crystallogr. 2009, 42, 339–341. 10.1107/S0021889811041161 PMC3236671 22199401

[30] Gaussian 09, Revision D.01, M. J. Frisch, G. W. Trucks, H. B. Schlegel, G. E. Scuseria, M. A. Robb, J. R. Cheeseman, G. Scalmani, V. Barone, G. A. Petersson, H. Nakatsuji, X. Li, M. Caricato, A. V. Marenich, J. Bloino, B. G. Janesko, R. Gomperts, B. Mennucci, H. P. Hratchian, J. V. Ortiz, A. F. Izmaylov, J. L. Sonnenberg, Williams, F. Ding, F. Lipparini, F. Egidi, J. Goings, B. Peng, A. Petrone, T. Henderson, D. Ranasinghe, V. G. Zakrzewski, J. Gao, N. Rega, G. Zheng, W. Liang, M. Hada, M. Ehara, K. Toyota, R. Fukuda, J. Hasegawa, M. Ishida, T. Nakajima, Y. Honda, O. Kitao, H. Nakai, T. Vreven, K. Throssell, J. A. Montgomery, Jr., J. E. Peralta, F. Ogliaro, M. J. Bearpark, J. J. Heyd, E. N. Brothers, K. N. Kudin, V. N. Staroverov, T. A. Keith, R. Kobayashi, J. Normand, K. Raghavachari, A. P. Rendell, J. C. Burant, S. S. Iyengar, J. Tomasi, M. Cossi, J. M. Millam, M. Klene, C. Adamo, R. Cammi, J. W. Ochterski, R. L. Martin, K. Morokuma, O. Farkas, J. B. Foresman, D. J. Fox, Wallingford, CT, **2016**.

[31a] C. Adamo , V. Barone , J. Chem. Phys. 1999, 110, 6158–6170;

[31b] M. Ernzerhof , G. E. Scuseria , J. Chem. Phys. 1999, 110, 5029–5036;

[31c] J. P. Perdew , K. Burke , M. Ernzerhof , Phys. Rev. Lett. 1996, 77, 3865–3868; 10062328 10.1103/PhysRevLett.77.3865

[31d] J. P. Perdew , M. Ernzerhof , K. Burke , J. Chem. Phys. 1996, 105, 9982–9985;

[31e] J. P. Perdew , K. Burke , M. Ernzerhof , Phys. Rev. Lett. 1997, 78, 1396–1396. 10.1103/PhysRevLett.77.3865 10062328

[32] A. Schäfer , C. Huber , R. Ahlrichs , J. Chem. Phys. 1994, 100, 5829–5835.

[33a] S. Grimme , J. Antony , S. Ehrlich , H. Krieg , J. Chem. Phys. 2010, 132, 154104; 20423165 10.1063/1.3382344

[33b] S. Grimme , S. Ehrlich , L. Goerigk , J. Comput. Chem. 2011, 32, 1456–1465. 21370243 10.1002/jcc.21759

[34] M. D. Hanwell , D. E. Curtis , D. C. Lonie , T. Vandermeersch , E. Zurek , G. R. Hutchison , J. Cheminf. 2012, 4, 17. 10.1186/1758-2946-4-17 PMC3542060 22889332

[35] E. D. Glendening, A. E. Reid, J. E. Carpenter, F. Weinhold, NBO Version 3.1.

