# Supplementary material for: A Xanthene‐Based Mono‐Anionic PON Ligand: Exploiting a Bulky, Electronically Unsymmetrical Donor in Main Group Chemistry
Source: Chemistry. 2021 Jan 14;27(9):3159–65. doi: 10.1002/chem.202004741 (PMC7898390; doi:10.1002/chem.202004741)
Supplement: Supplementary file 1 — Supplementary [file CHEM-27-3159-s001.pdf]

Chemistry–A European Journal

Supporting Information

A Xanthene-Based Mono-Anionic PON Ligand: Exploiting a Bulky, Electronically Unsymmetrical Donor in Main Group Chemistry

Xiongfei Zheng, Andreas Heilmann, Caitilín McManus, and Simon Aldridge*[a]

Supporting Information

Table of contents

1. Additional synthetic and characterizing data s2

2. ^{1}H, $^{31}P\{^{1}H\}$, $^{13}C\{^{1}H\}$ and ^{19}F NMR spectra of novel compounds **2**-**7** and (PON)GaI_2 s3

3. xyz Coordinates for the DFT optimized structures of amidogallium systems s25

4. Crystallographic data s33

1. Additional synthetic and characterizing data

H(PON), 2: To a mixture of **1** (5.00 g, 9 mmol), NaO^{t}Bu (2.60 g, 27 mmol), Pd(OAc)$_2$ (0.04 g, 0.18 mmol) and DPEPhos (0.15 g, 0.27 mmol), in toluene (50 mL) was added 2,6-diisopropylaniline (1.9 mL, 10 mmol) at room temperature. The reaction mixture was heated to 100 ˚C overnight whereupon it was quenched with water and extracted with toluene (3 x 25 mL). After drying over $MgSO_4$ and removal of volatiles *in vacuo* a brown solid was obtained, which was recrystalized from toluene/ methanol and dried to give **2** as a white powder (4.20 g, 72 %). Single crystals suitable for X-ray crystallography were obtained by slow evaporation from a concentrated solution in toluene at room temperature. ^{1}H NMR (400 MHz, C_6D_6, 298 K): δ = 1.05 (d, J_{HH} = 6.9 Hz, 6H, CH(CH_3)$_2$), 1.14 (d, J_{HH} = 6.9 Hz, 6H, CH(CH_3)$_2$), 1.46 (s, 6H, CH_3 of XA), 2.09 (s, 6H, *p*-CH_3 of Mes), 2.33 (s, 12H, *o*-CH_3 of Mes), 3.33 (sept, J_{HH} = 6.9 Hz, 2H, C*H*(CH$_3$)$_2$), 6.03 (s, 1H, N*H*), 6.19 (dd, J_{HH} = 1.2, 8.0 Hz, 1H, Ar*H*), 6.66 (dd, J_{HH} = 1.2, 8.0 Hz, 1H, Ar*H*), 6.71 (d, J_{HH} = 2.8 Hz, 4H, C*H* of Mes), 6.77 (dd, J_{HH} = 1.0, 7.6 Hz, 1H, Ar*H*), 6.8 (d, J_{HH} = 8.0 Hz, 1H, Ar*H*), 7.07 (m, 1H, Ar*H*), 7.11 (m, 1H, Ar*H*), 7.18 (m, 3H, Ar*H* of Dipp) ppm. ^{13}C NMR (126 MHz, C_6D_6, 298 K): δ = 153.5 (d, J_{PC} = 19.3 Hz), 148.4, 143.7 (d, J_{PC} = 16.2 Hz), 138.4, 138.1 (d, J_{PC} = 2.3 Hz), 137.5, 135.6, 130.8, 130.6 (d, J_{PC} = 3.8 Hz), 130.4, 129.9, 129.8, 129.7, 126.4, 126.1 (d, J_{PC} = 13.6 Hz), 124.1, 124.0, 123.8, 113.4, 109.5, 34.7 (d, J_{PC} = 1.8 Hz), 31.5, 28.6, 24.9, 23.2, 22.8, 22.6, 21.0 ppm. ^{31}P NMR (162 MHz, C_6D_6, 298 K): δ = -36.9 ppm.

(PON)GaI$_2$: A mixture of Ga metal (12 mg, 0.16 mmol) and iodine (63 mg, 0.25 mmol) in toluene (5 mL) was sonicated until the solution turned colourless. A solution of **3** (0.11 g, 0.17 mmol) also in toluene (5 mL) was then transferred dropwise into the GaI_3 solution. After stirring at room temperature overnight, the reaction mixture was allowed to settle and filtered by cannula. The resulting filtrate was concentrated (to ca. 3 mL) and crystalline product obtained from slow evaporation at room temperature. (0.13 g, 81 %). ^{1}H NMR (400 MHz, C_6D_6, 343 K): δ = 1.01 (s, 6H, CH(CH_3)$_2$), 1.26 (s, 6H, CH(CH_3)$_2$), 1.56 (s, 6H, CH_3 of XA), 1.99 (s, 6H, *p*-CH_3 of Mes), 2.26 (br, 12H, *o*-CH_3 of Mes), 3.14 (s, 2H, C*H*(CH$_3$)$_2$), 6.20 (dd, J_{HH} = 1.1, 8.0 Hz, 1H, Ar*H*), 6.53 (dd, J_{HH} = 1.3, 7.9 Hz, 1H, Ar*H*), 6.60 (s, 4H, C*H* of Mes), 6.71 (m, 1H, Ar*H*), 6.83 (m, 2H, Ar*H*), 7.23 (d, J_{HH} = 7.9 Hz, 1H, *p*-C*H* of Dipp), 7.29 (m, 2H, *m*-C*H* of Dipp) ppm. ^{13}C NMR (126 MHz, C_6D_6, 343 K): δ = 149.7 (d, J_{PC} = 6.9 Hz), 149.5, 143.4, 141.6, 141.2, 141.0, 134.0 (d, J_{PC} = 4.2 Hz), 132.6, 131.9 (d, J_{PC} = 7.4 Hz), 131.8, 131.6, 128.6, 127.3, 125.2, 124.7 (d, J_{PC} = 7.4 Hz), 124.6, 116.9, 116.6, 114.6, 112.7, 34.5, 33.4, 29.1, 25.3, 20.7 ppm. ^{31}P NMR (162 MHz, C_6D_6, 298 K): δ = -30.8 ppm.

2. ^{1}H, $^{31}P\{^{1}H\}$, $^{13}C\{^{1}H\}$ and ^{19}F NMR spectra of novel compounds 2-7 and $(PON)GaI_2$

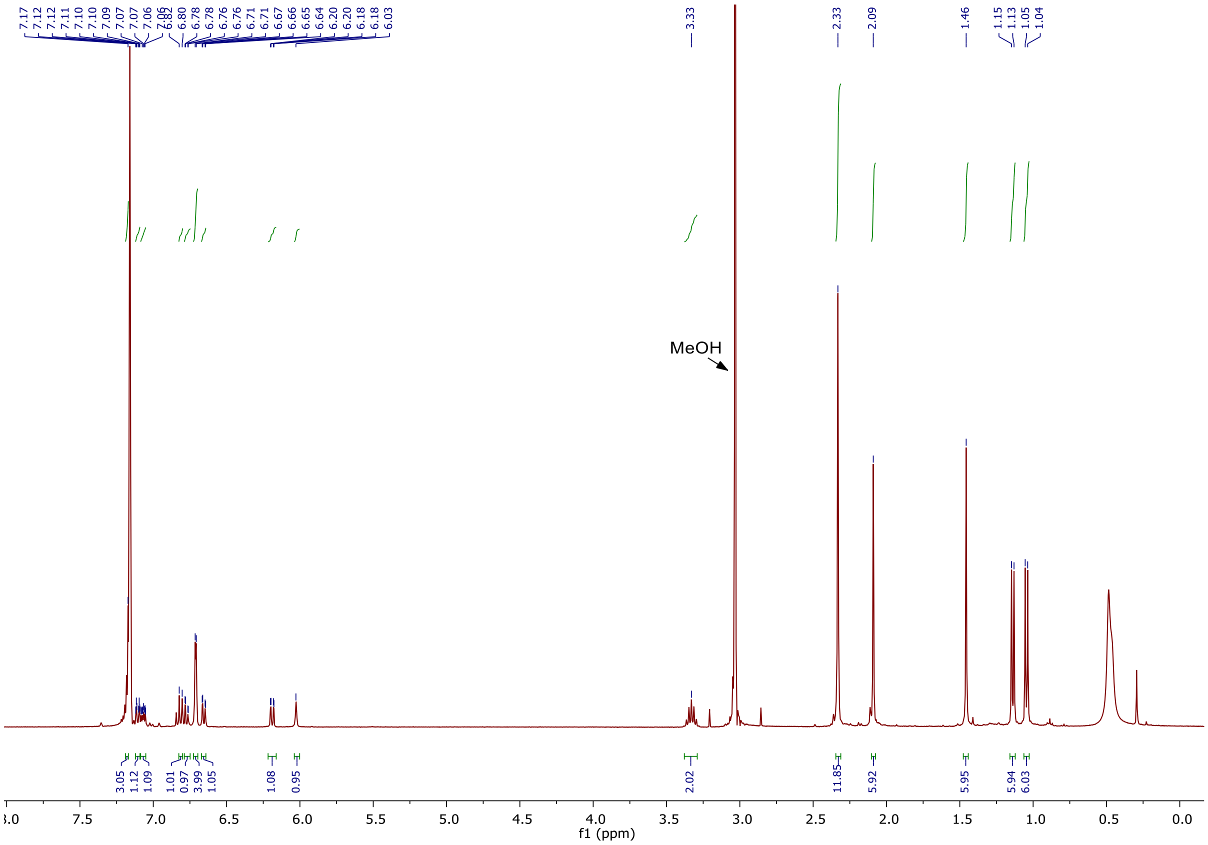

Figure s1. ^{1}H NMR of H(PON) (**2**) in C_6D_6 at 298 K.

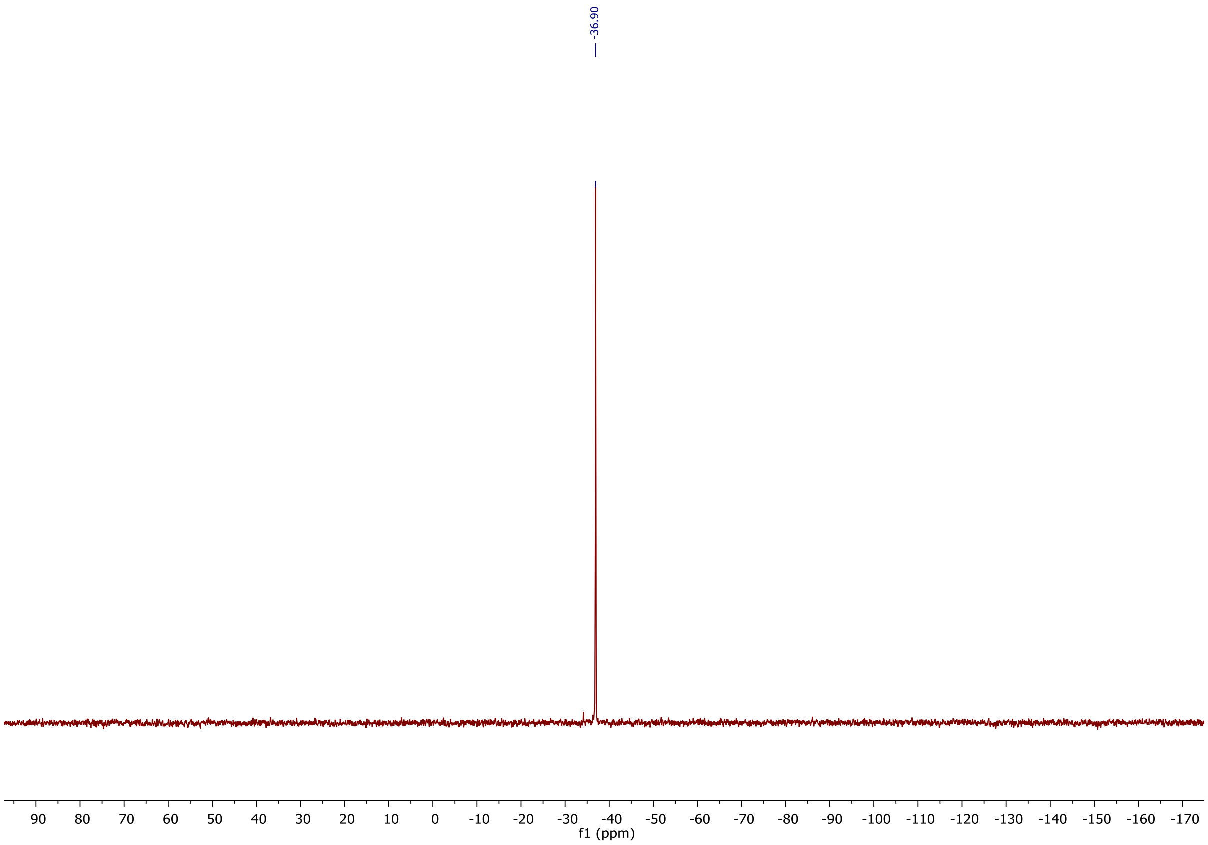

Figure s2. $^{31}P\{^{1}H\}$ NMR spectrum of H(PON) (**2**) in C_6D_6 at 298 K.

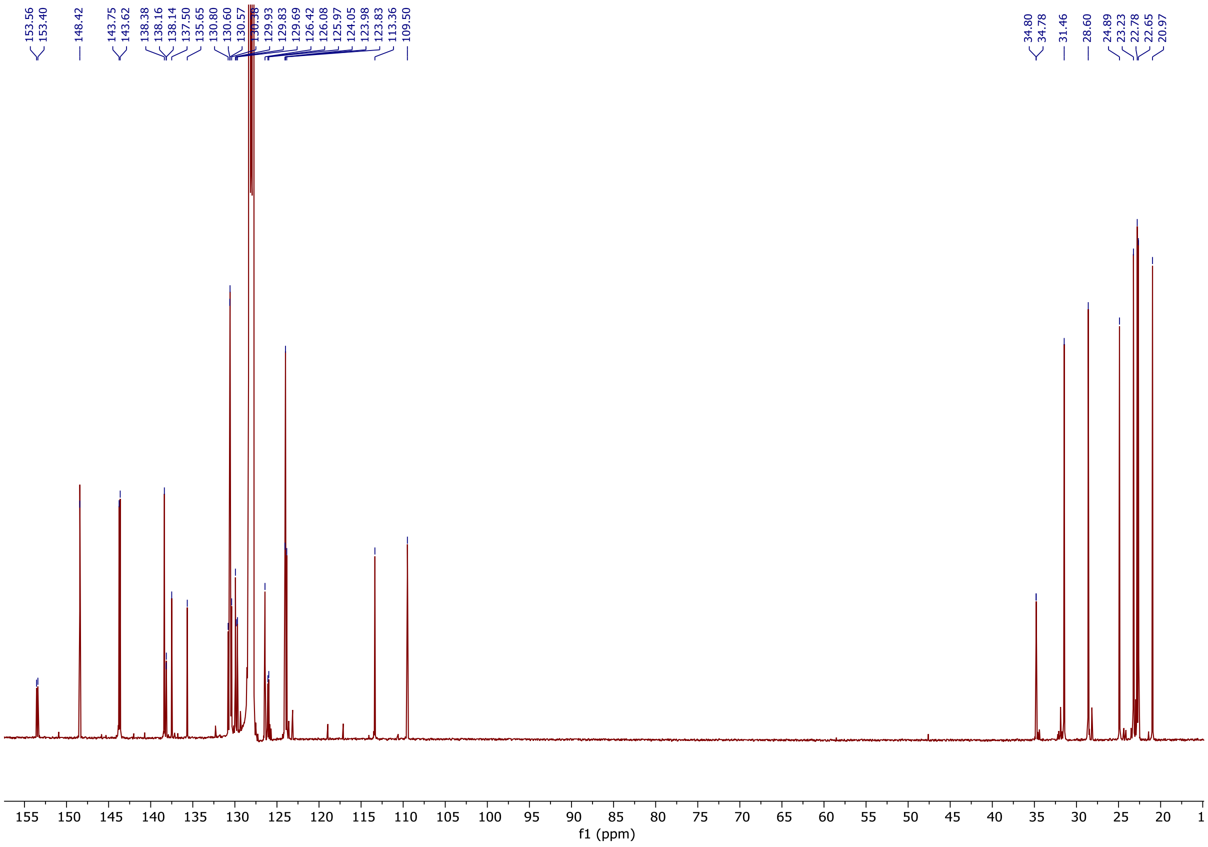

Figure s3. $^{13}C\{^{1}H\}$ NMR spectrum of H(PON) (**2**) in C_6D_6 at 298 K.

[K(PON)]$_2$, **3**

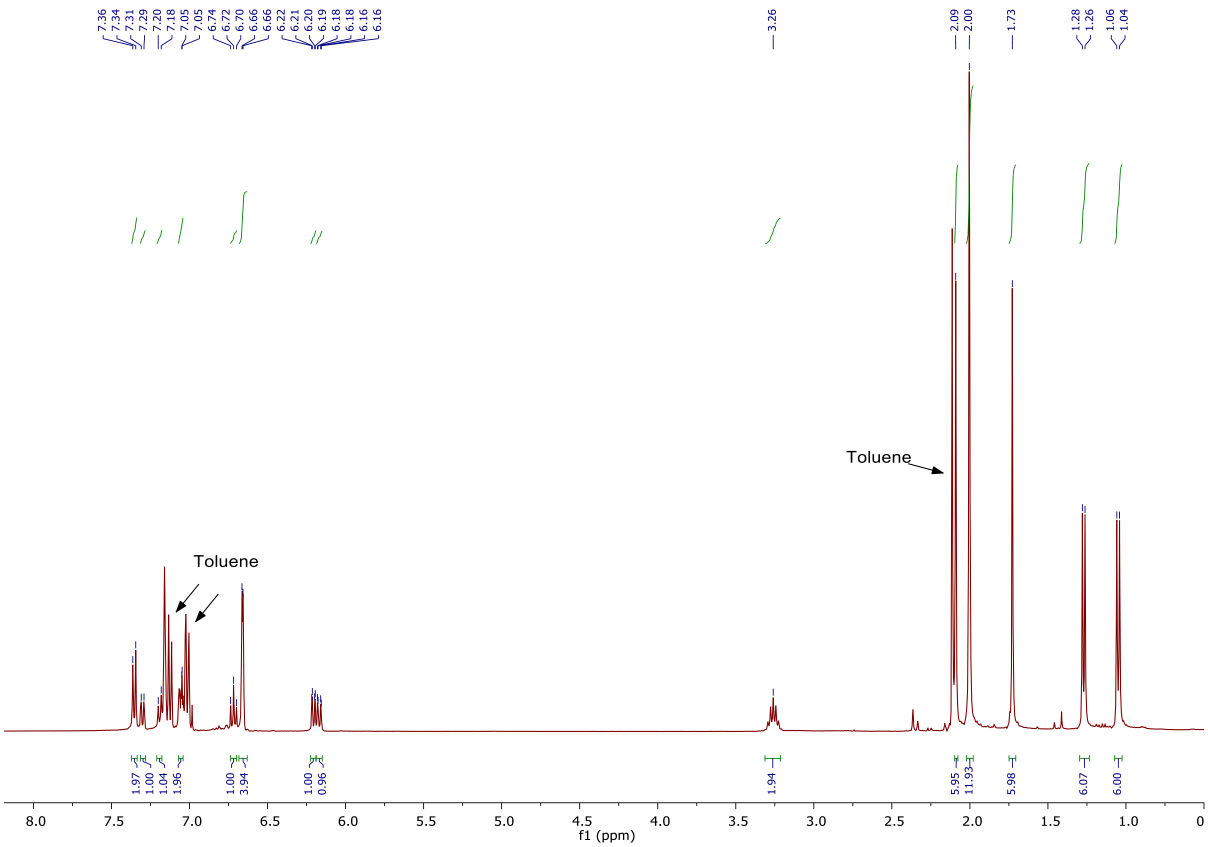

Figure s4. ^{1}H NMR spectrum of [K(PON)]$_2$ (**3**) in C_6D_6 at 298 K.

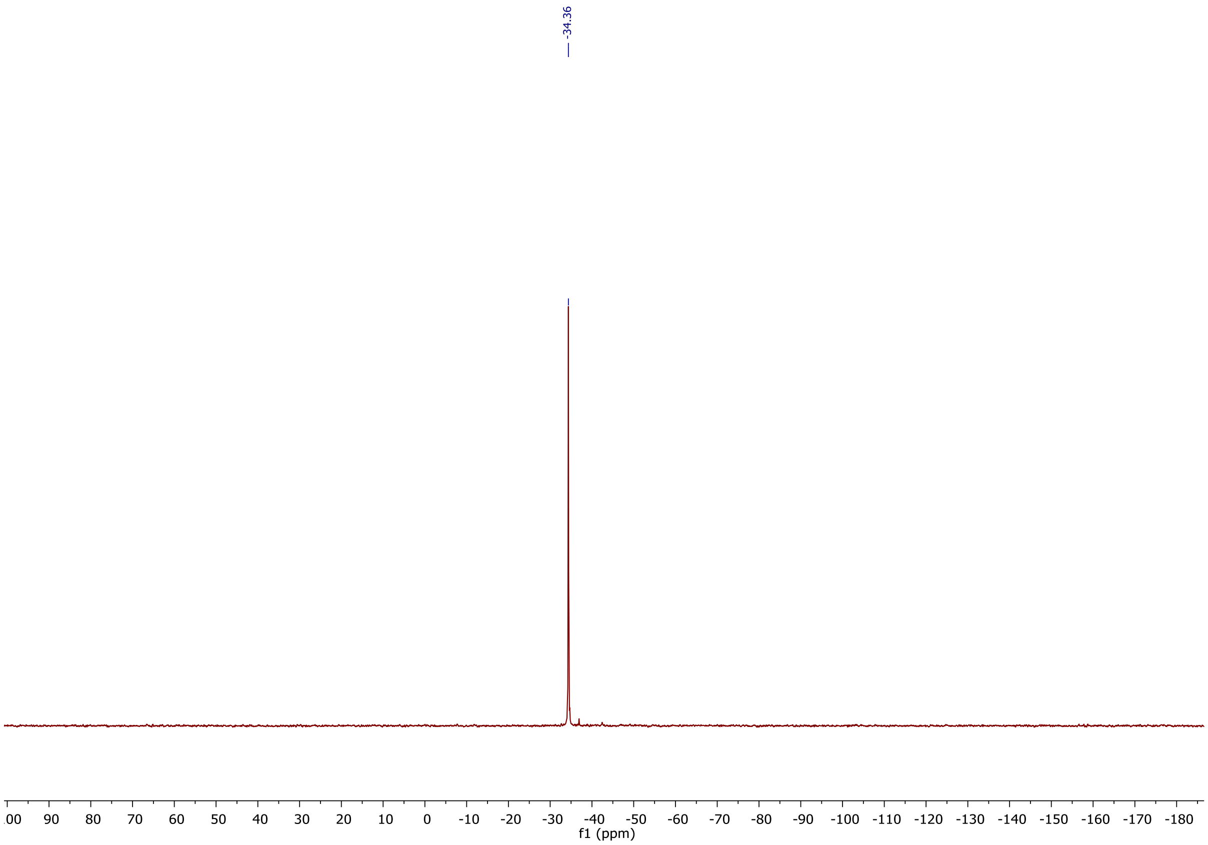

Figure s5. $^{31}P\{^1H\}$ NMR spectrum of $[K(PON)]_2$ (**3**) in C_6D_6 at 298 K.

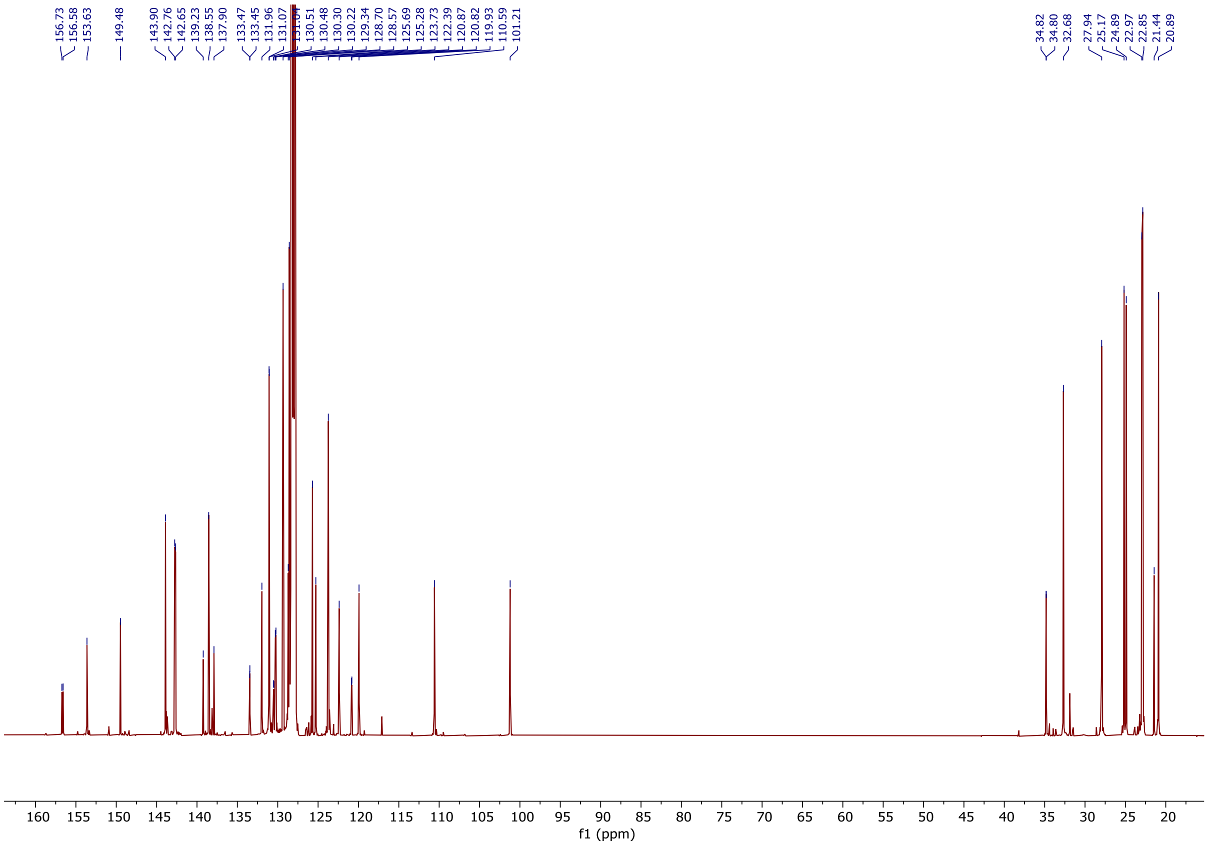

Figure s6. $^{13}C\{^{1}H\}$ NMR of compound **3** KPON in C_6D_6 at 298K.

(PON)Ga, **4**

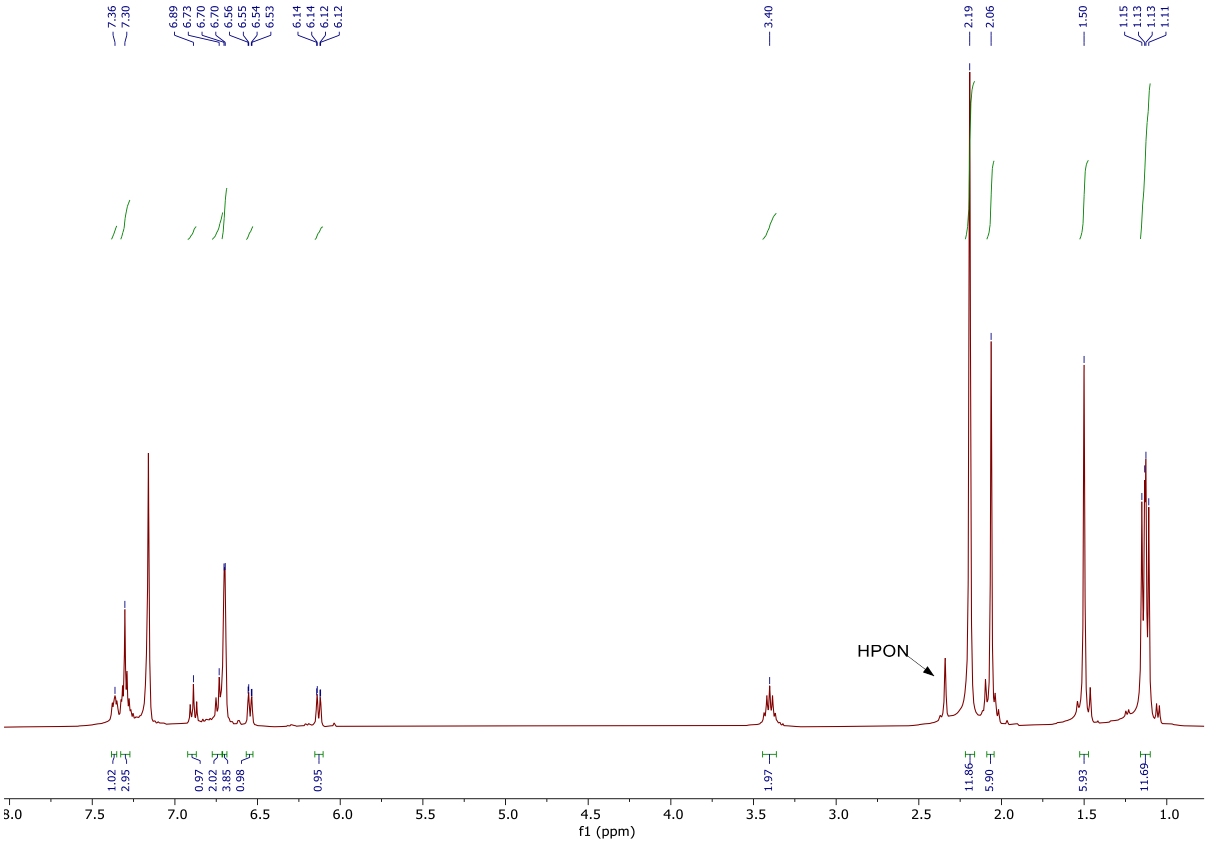

Figure s7. ^{1}H NMR spectrum of (PON)Ga (**4**) in C_6D_6 at 298 K.

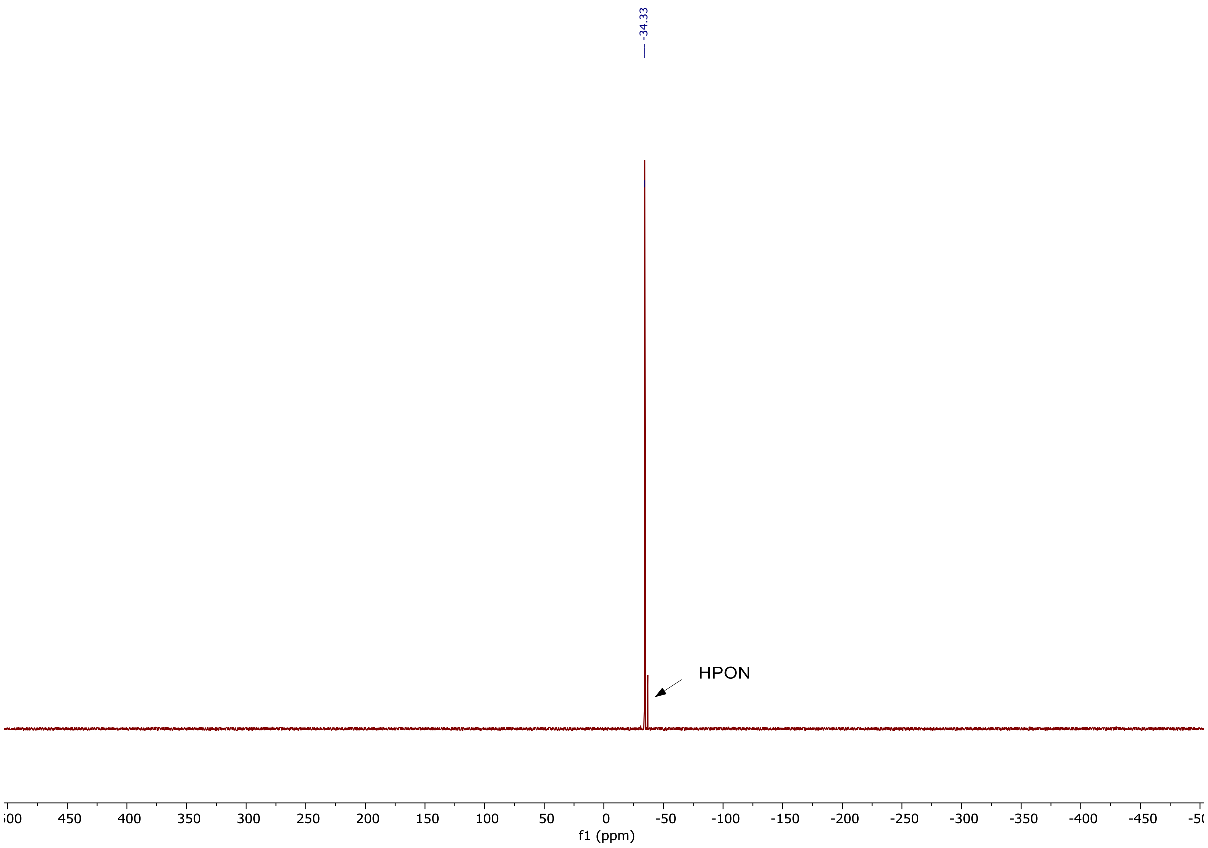

Figure s8. $^{31}P\{^1H\}$ NMR spectrum of (PON)Ga (**4**) in C_6D_6 at 298 K.

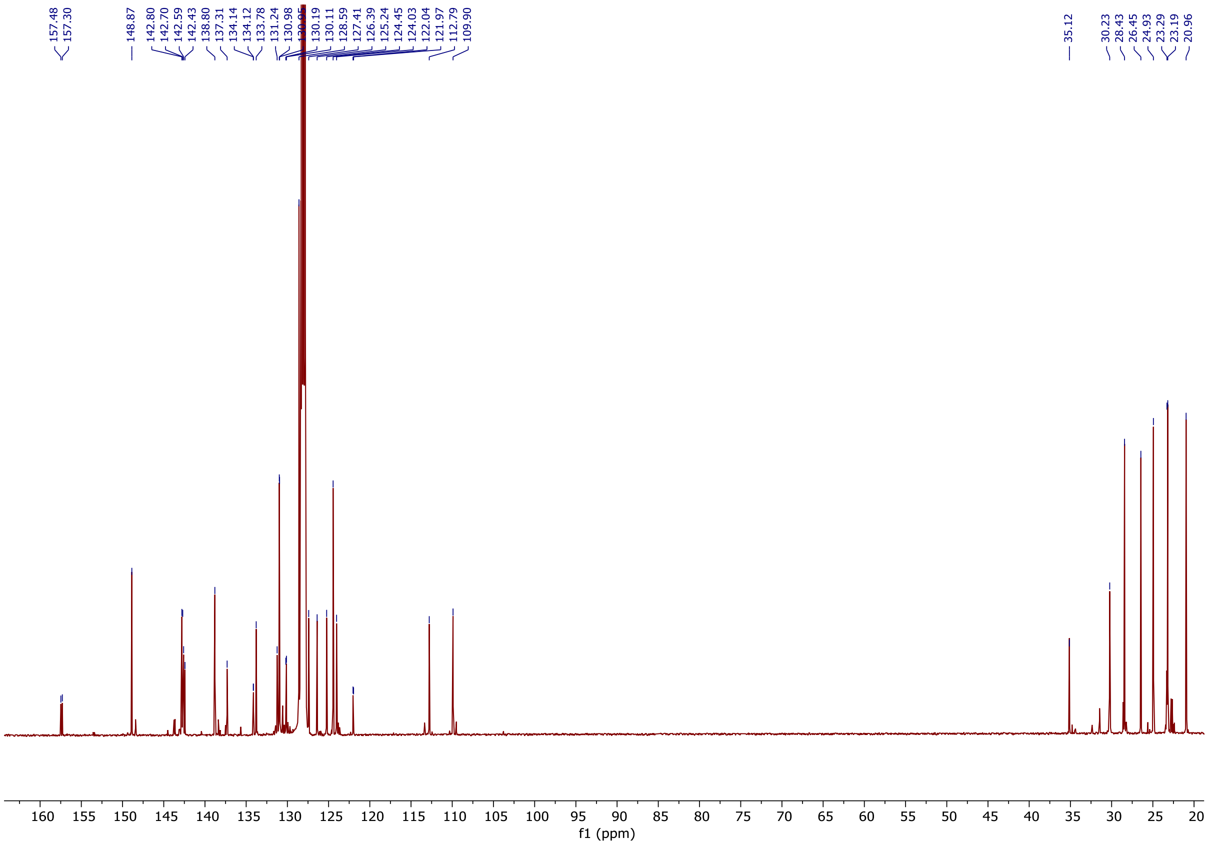

Figure s9. $^{13}C\{^1H\}$ NMR spectrum of (PON)Ga (**4**) in C_6D_6 at 298 K.

(PON)GaI_2

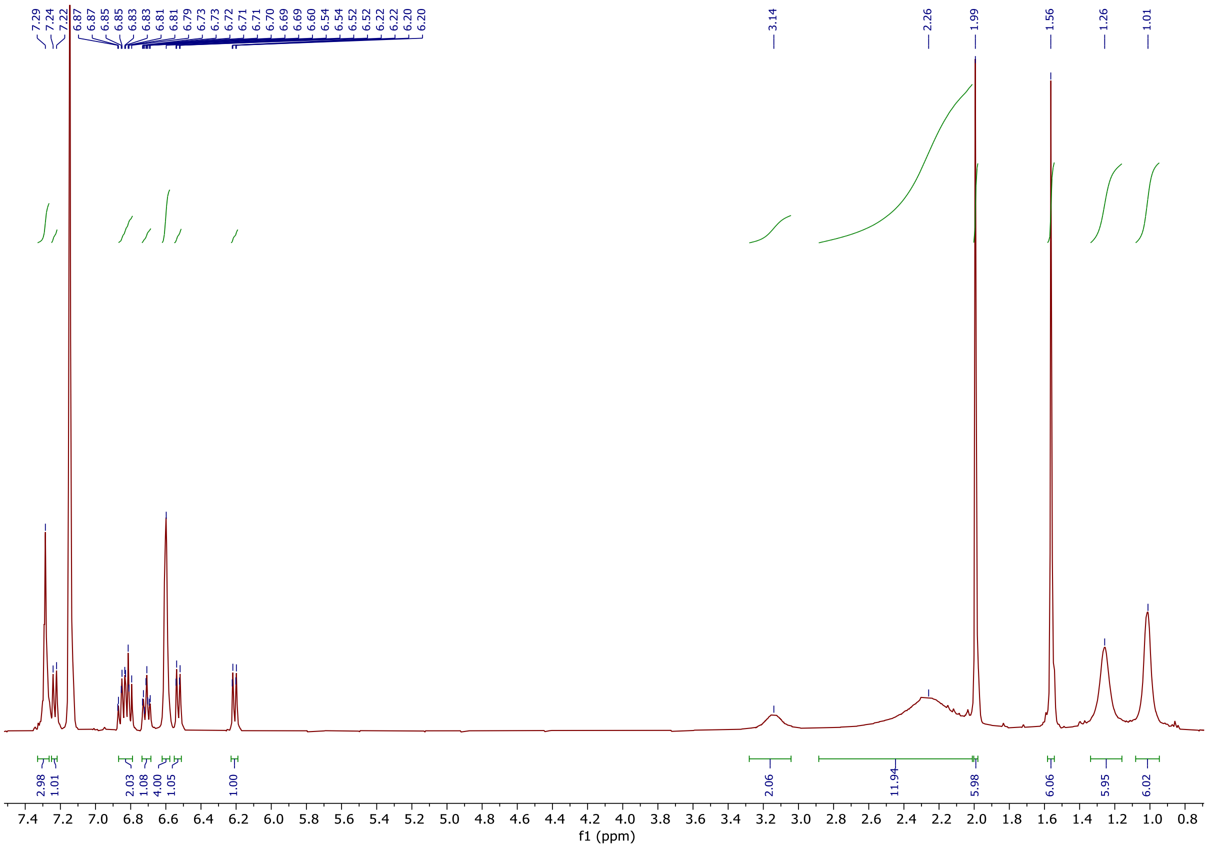

Figure s10. ^{1}H NMR spectrum of (PON)GaI_2 in C_6D_6 at 343 K.

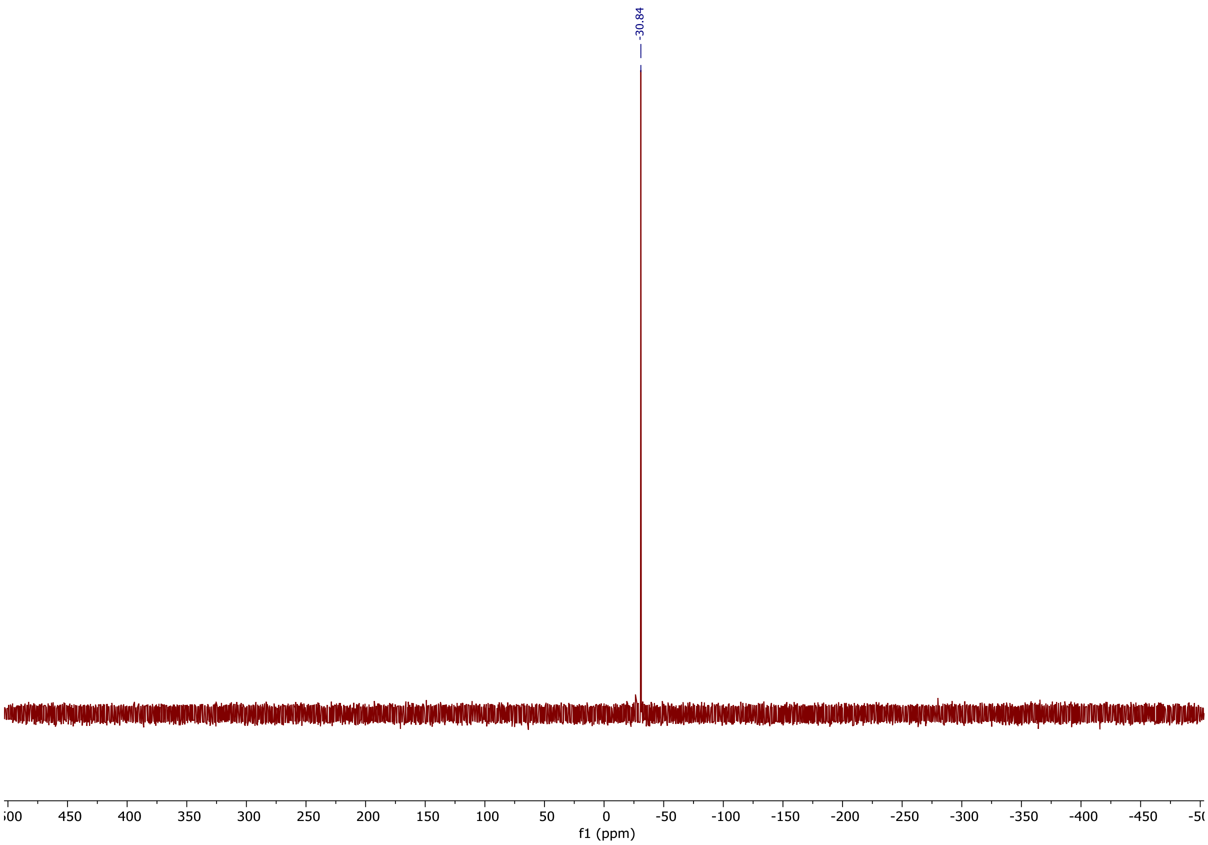

Figure s11. $^{31}P\{^{1}H\}$ NMR spectrum of (PON)GaI_2 in C_6D_6 at 298 K.

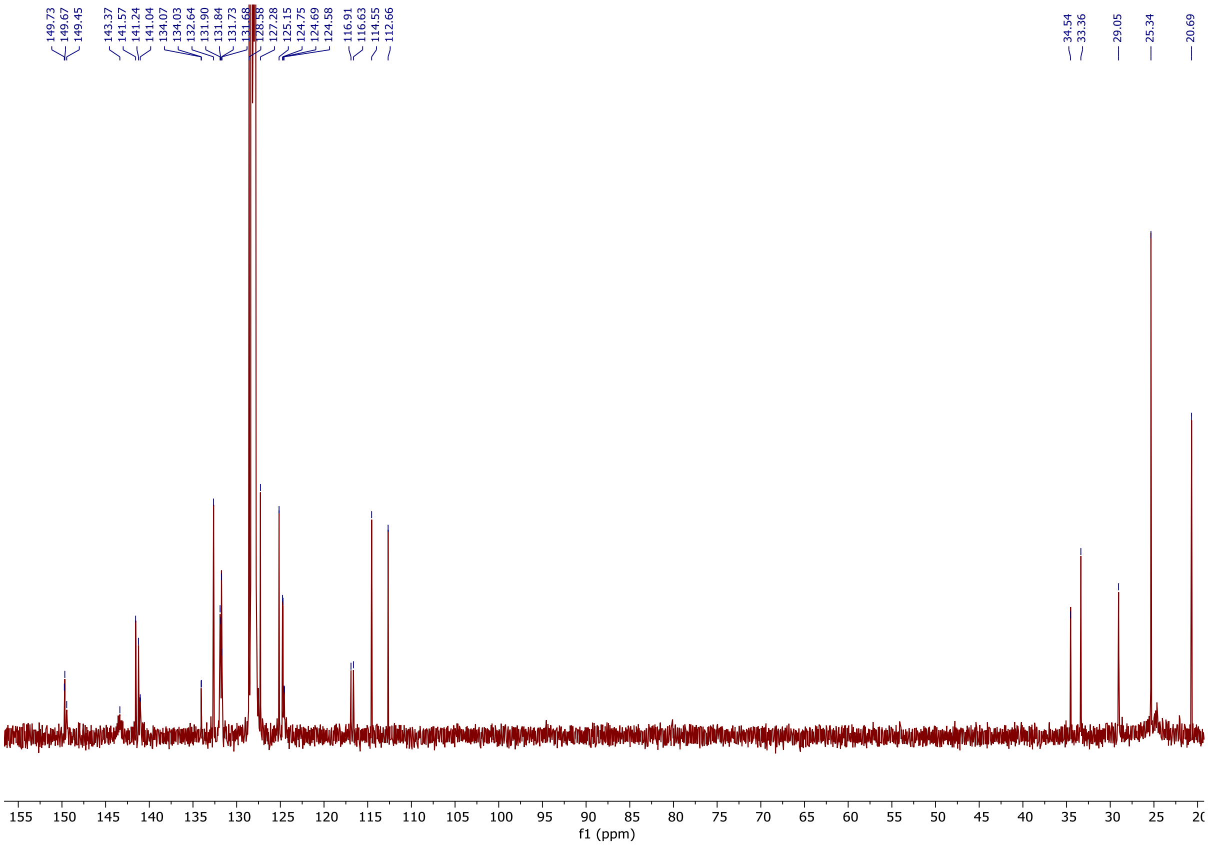

Figure s12. $^{13}C\{^{1}H\}$ NMR spectrum of (PON)GaI_2 in C_6D_6 at 343 K.

CH activation product **5**

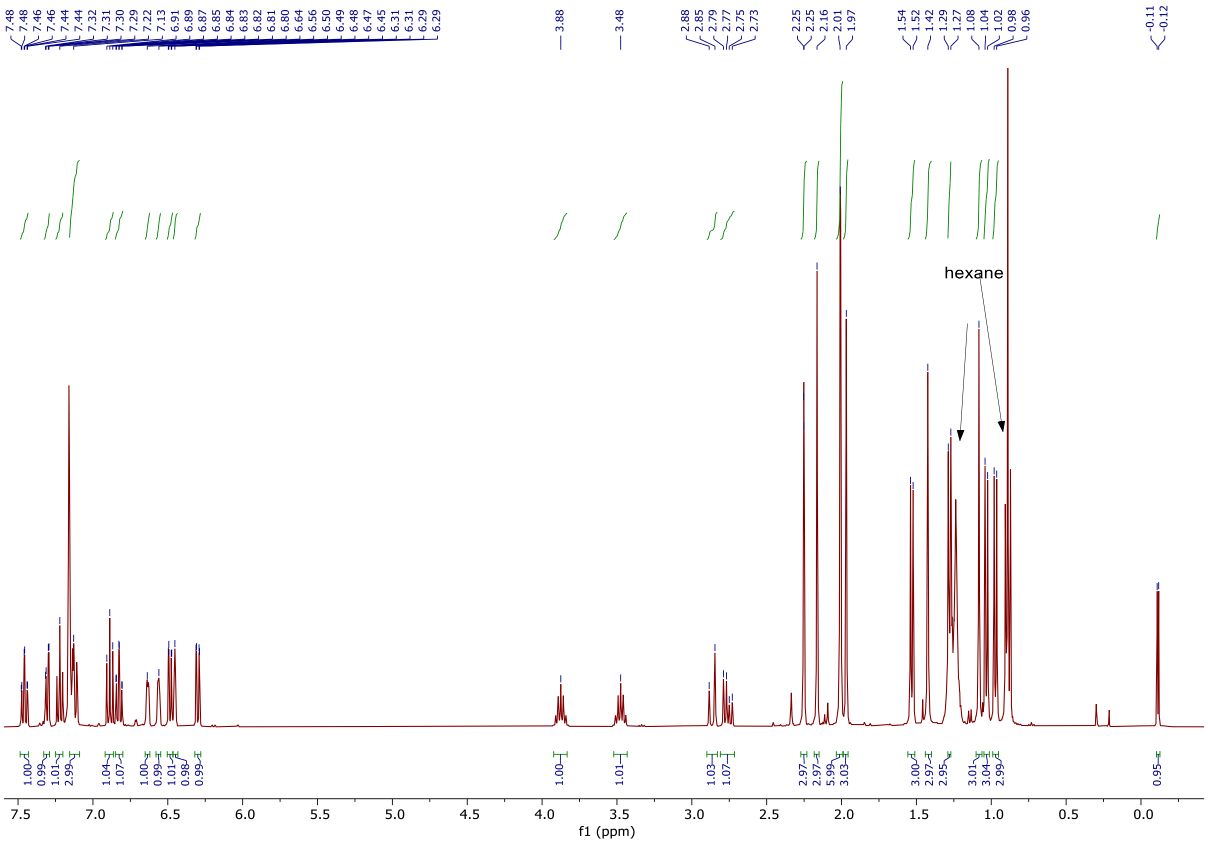

Figure s13. ^{1}H NMR spectrum of compound **5** in C_6D_6 at 298 K.

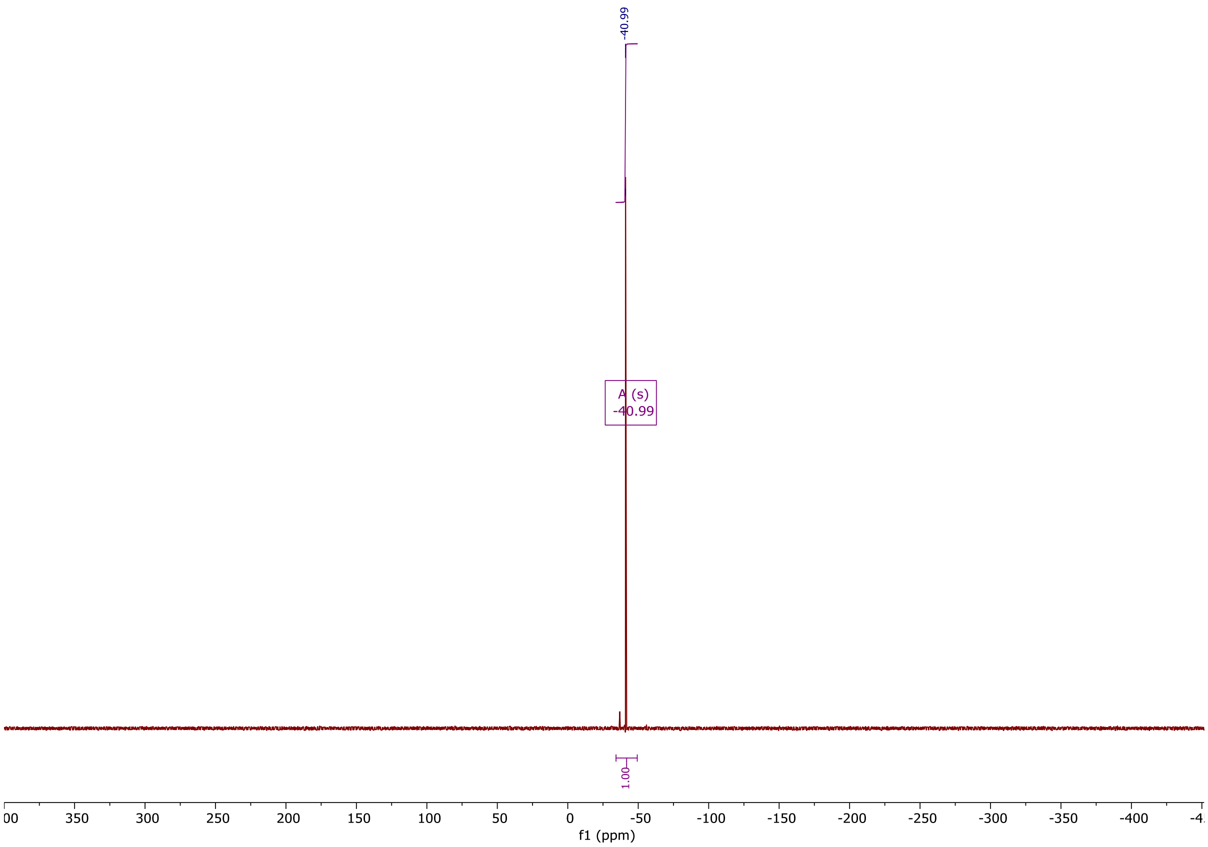

Figure s14 $^{31}P\{^{1}H\}$ NMR spectrum of compound **5** in C_6D_6 at 298 K.

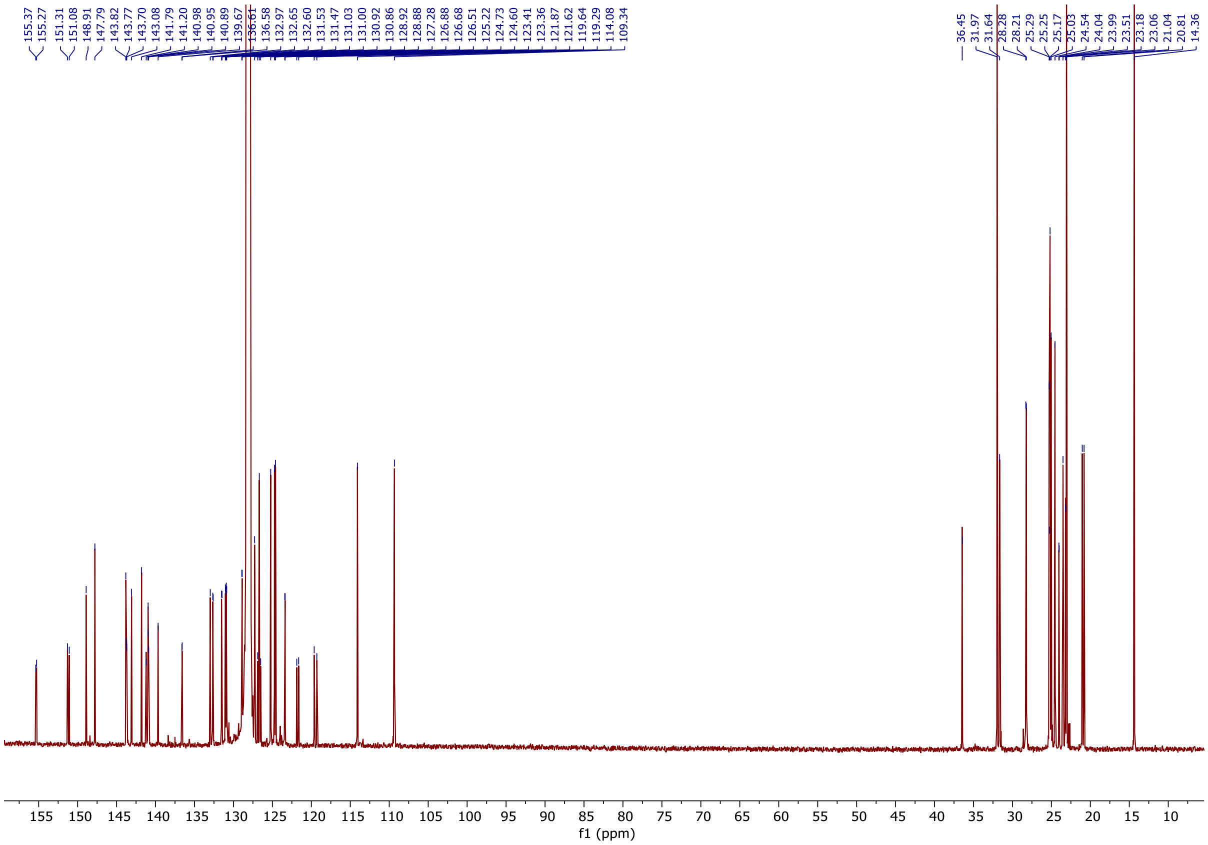

Figure s15. $^{13}C\{^1H\}$ NMR spectrum of compound **5** in C_6D_6 at 298 K.

BPh$_3$ activation product **6**

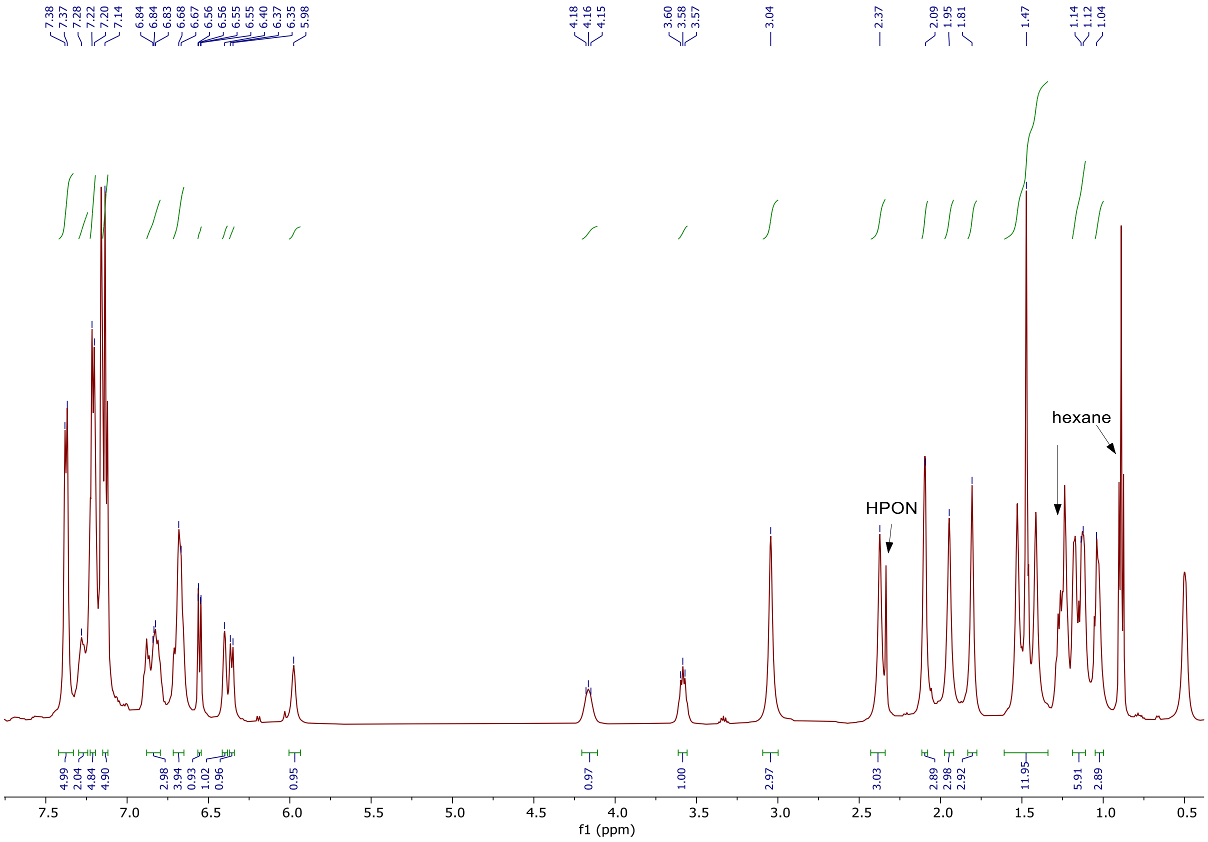

Figure s16. ^{1}H NMR spectrum of compound **6** in C_6D_6 at 298 K.

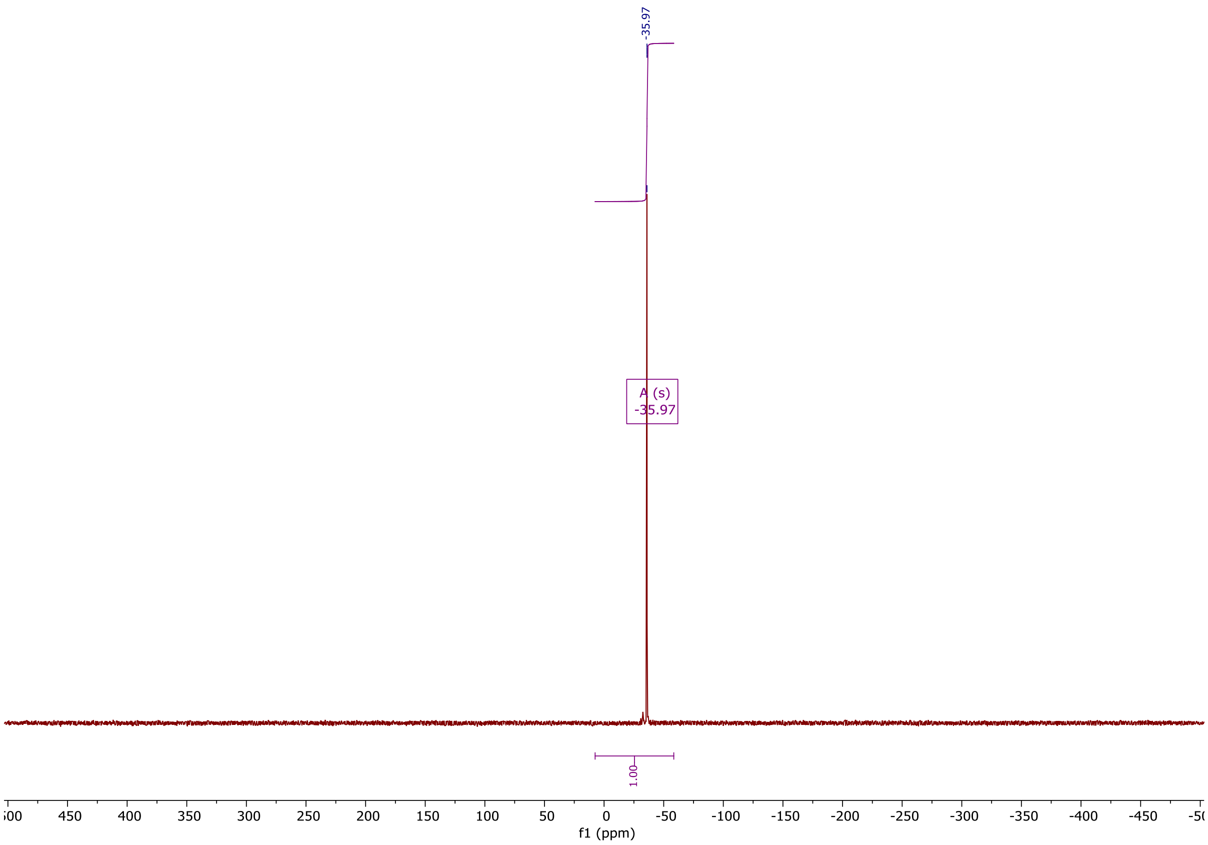

Figure s17. $^{31}P\{^{1}H\}$ NMR spectrum of compound **6** in C_6D_6 at 298 K.

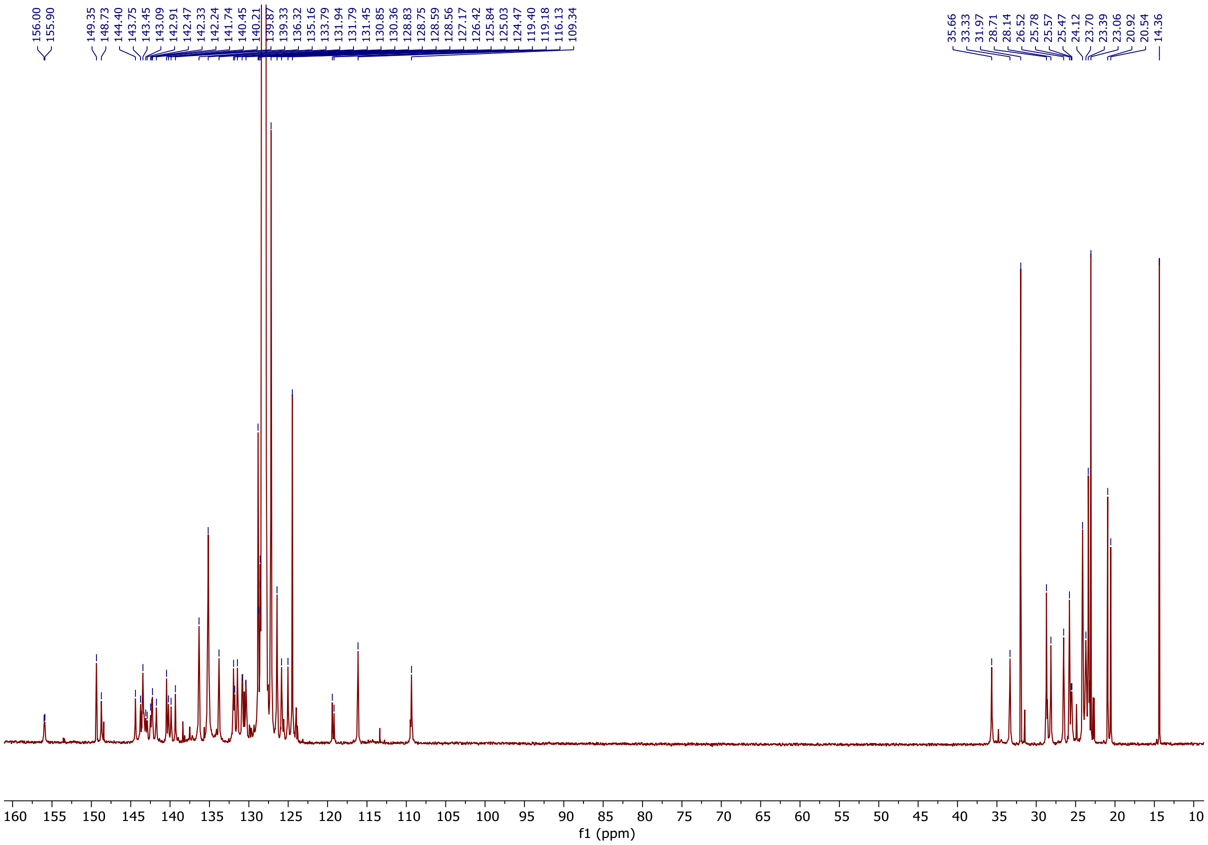

Figure s18. $^{13}C\{^{1}H\}$ NMR spectrum of compound **6** in C_6D_6 at 298 K.

B(C_6F_5)$_3$ activation product **7**

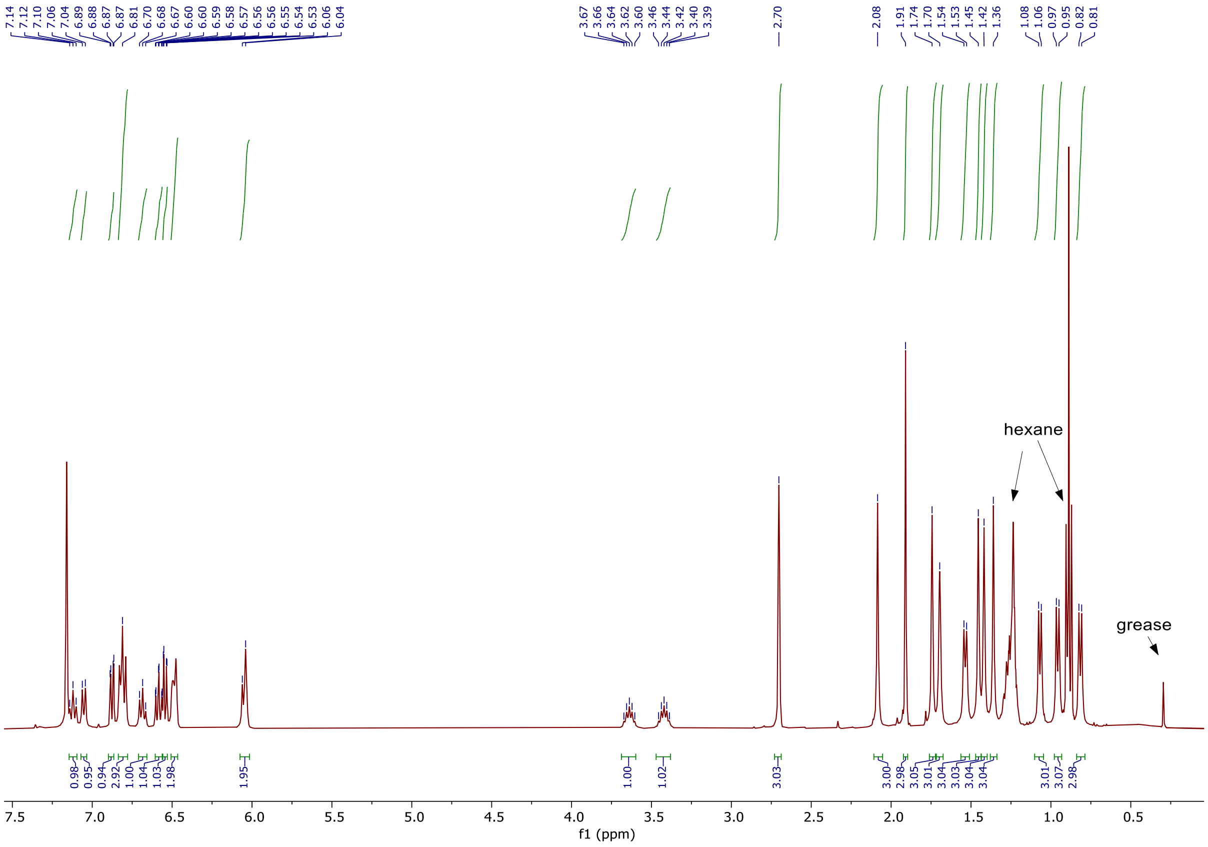

Figure s19. ^{1}H NMR spectrum of compound **7** in C_6D_6 at 298 K.

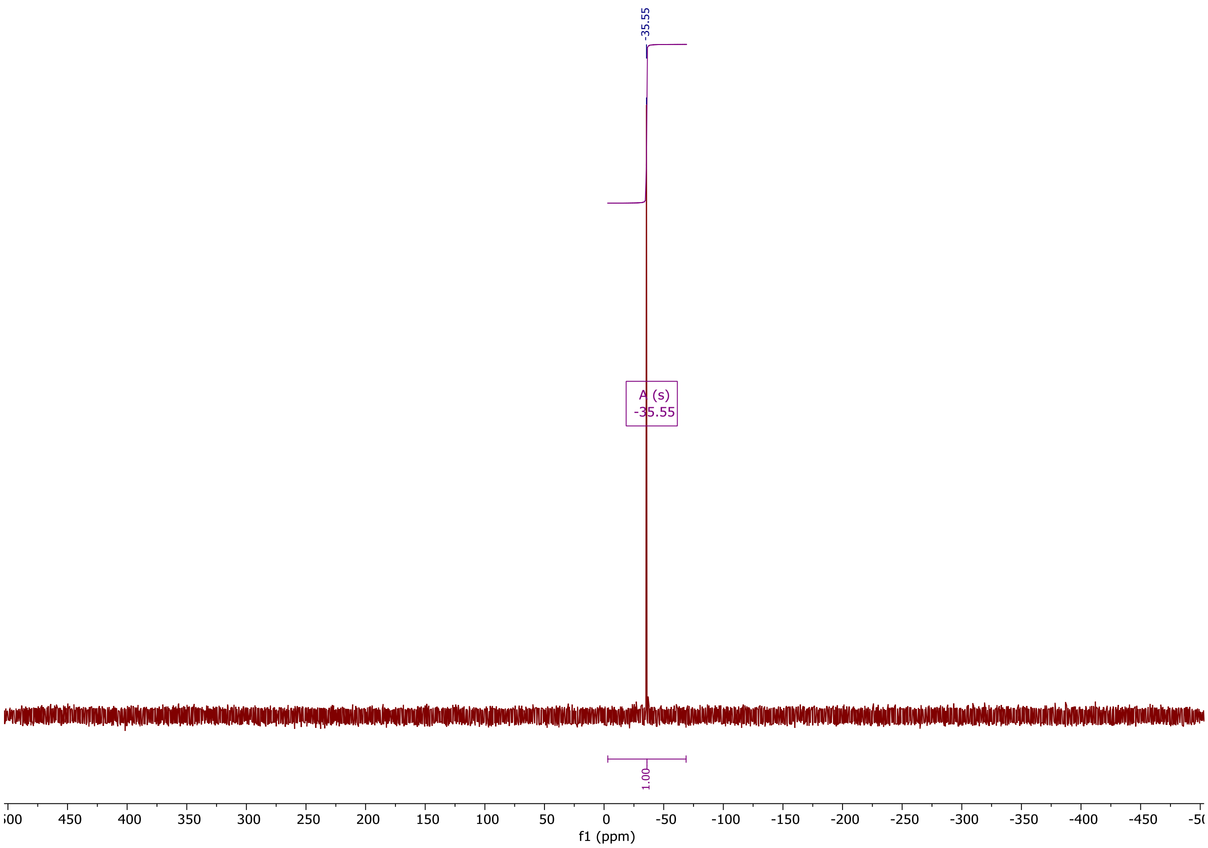

Figure s20. $^{31}P\{^1H\}$ NMR spectrum of compound **7** in C_6D_6 at 298 K.

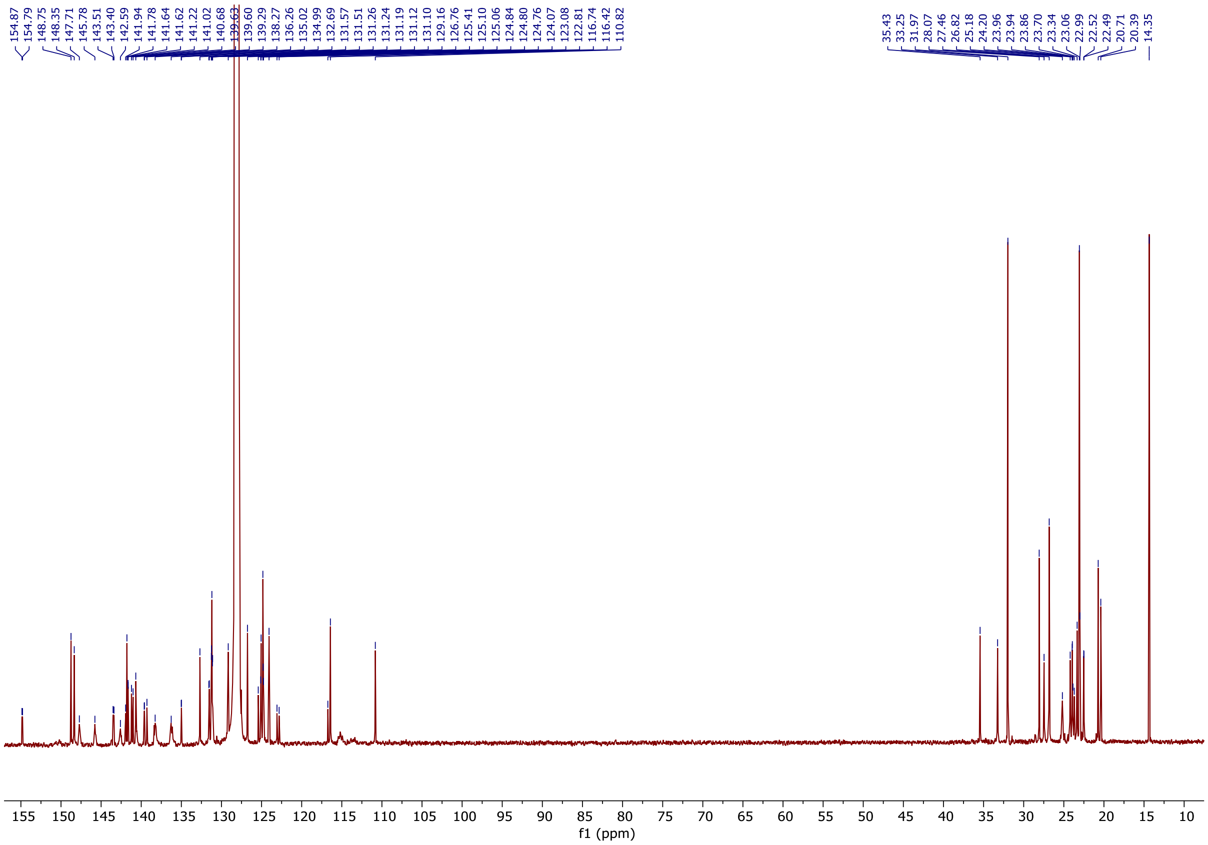

Figure s21. $^{13}C\{^{1}H\}$ NMR spectrum of compound **7** in C_6D_6 at 298 K.

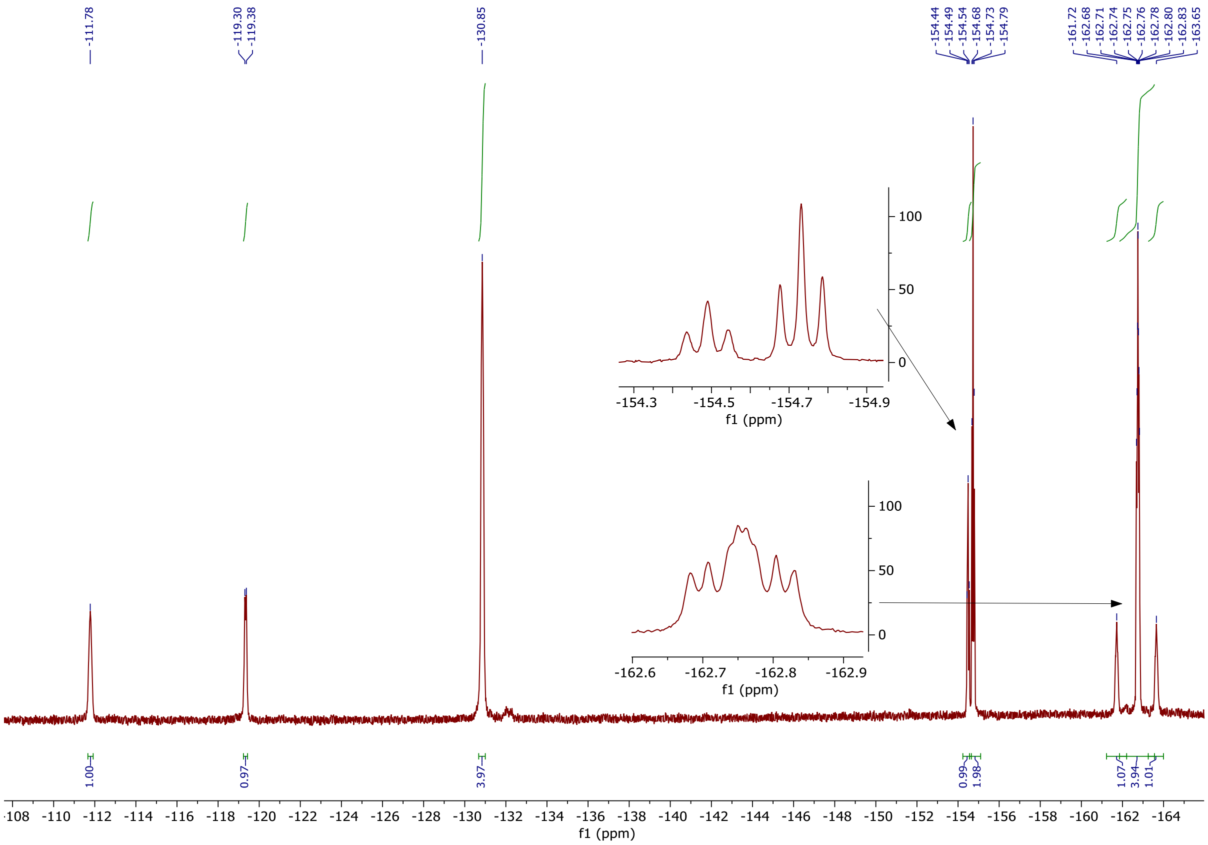

Figure s22. ^{19}F NMR spectrum of compound **7** in C_6D_6 at 298 K.

3. xyz Coordinates for the DFT optimized structures of amidogallium systems:

NacnacGa
73

Ga	-0.95575	9.36878	10.12796
N	0.04641	10.81290	9.00246
C	0.16149	12.11196	9.23866
N	-1.45806	10.89937	11.45351
C	-0.40330	12.75149	10.34641
H	-0.23267	13.81700	10.41386
C	-1.15572	12.18794	11.38140
C	0.94125	12.97189	8.28177
H	1.98246	12.64374	8.23381
H	0.91592	14.01760	8.58450
H	0.54157	12.88448	7.26914
C	-1.63262	13.12024	12.46140
H	-1.23162	12.81918	13.43219
H	-2.72068	13.08268	12.55031
H	-1.32869	14.14580	12.25728
C	0.66555	10.25074	7.84603
C	1.97892	9.75565	7.93441
C	2.53250	9.13577	6.81845
H	3.54423	8.74809	6.87309
C	1.81530	8.99821	5.64297
H	2.26311	8.50956	4.78452
C	0.52217	9.48485	5.57086
H	-0.03954	9.37071	4.64956
C	-0.07597	10.11288	6.65946
C	-1.50328	10.60894	6.54090
H	-1.73751	11.16275	7.45304
C	-2.48314	9.43853	6.44545
H	-2.38862	8.76994	7.30474
H	-2.29923	8.84820	5.54323
H	-3.51436	9.80128	6.40526
C	-1.68927	11.56347	5.36207
H	-0.99558	12.40645	5.41067
H	-2.70691	11.96328	5.35493
H	-1.52939	11.05787	4.40600
C	2.78724	9.85342	9.21284
H	2.24533	10.50934	9.89825
C	2.90625	8.48497	9.88527
H	1.92155	8.05780	10.09221
H	3.45070	8.56477	10.83068
H	3.44489	7.78129	9.24400
C	4.16939	10.46386	8.98585
H	4.10651	11.43720	8.49278
H	4.79845	9.81929	8.36613
H	4.68421	10.60121	9.94060
C	-2.22576	10.41803	12.55632
C	-1.56901	9.96314	13.71379
C	-2.33329	9.41885	14.74115
H	-1.83953	9.06257	15.63902

C	-3.70962	9.31721	14.63717
H	-4.28760	8.88732	15.44795
C	-4.34301	9.76451	13.49139
H	-5.42174	9.67951	13.41141
C	-3.62286	10.31626	12.43591
C	-4.35693	10.77260	11.19065
H	-3.63197	11.27501	10.54581
C	-5.46523	11.77610	11.50621
H	-5.08455	12.63745	12.06062
H	-6.26045	11.32308	12.10420
H	-5.91927	12.14264	10.58153
C	-4.91400	9.57884	10.41399
H	-5.40503	9.91253	9.49529
H	-5.65115	9.03278	11.00976
H	-4.12100	8.87727	10.14285
C	-0.06126	10.02698	13.85652
H	0.32364	10.63395	13.03359
C	0.55712	8.63398	13.72918
H	0.28340	8.16245	12.78183
H	0.21383	7.97991	14.53584
H	1.64831	8.68997	13.78204
C	0.37548	10.69189	15.16119
H	1.46307	10.80169	15.18398
H	0.08985	10.09762	16.03325
H	-0.06675	11.68444	15.27825

$[(NON)Ga]^-$

113

Ga	12.19872	9.79195	10.33996
O	13.90790	10.32911	11.91348
N	13.25851	11.66302	9.78540
N	11.49956	9.49827	12.44232
C	14.57463	10.40786	14.20459
C	10.30175	8.79487	12.62860
C	13.63455	10.12220	13.24546
C	15.16627	13.20693	10.15068
H	14.94406	13.75775	9.24324
C	14.23211	10.17107	15.54259
H	14.95001	10.39422	16.31825
C	14.33998	12.11448	10.46923
C	12.94201	12.28265	8.56658
C	14.69865	11.42062	11.64055
C	15.93660	10.90886	13.72487
C	10.30395	7.38504	12.73771
C	12.98256	9.65125	15.87068
C	16.22269	13.60576	10.97421
C	12.05798	9.38710	14.85709
H	11.07382	9.00455	15.10379
C	12.33522	9.64433	13.50235
C	16.47874	12.90570	12.15033

H	17.27772	13.21799	12.80711
C	13.50034	11.80844	7.35823
C	12.01617	13.34801	8.52399
C	9.06544	9.48184	12.62243
C	15.70821	11.78672	12.49498
C	14.49189	10.66414	7.34765
H	14.64569	10.37259	8.38858
C	9.09754	6.70758	12.88473
H	9.10469	5.62445	12.96670
C	11.68027	13.91593	7.29871
H	10.97086	14.73817	7.27081
C	16.68340	11.65807	14.82272
H	17.66148	11.98651	14.46449
H	16.86000	11.00654	15.68138
H	16.12532	12.53425	15.16014
C	7.89015	7.38307	12.90855
H	6.95736	6.83953	13.02093
C	11.58883	6.58733	12.64336
H	12.41485	7.29979	12.64402
C	17.06974	14.81123	10.55390
C	13.13210	12.40277	6.15508
H	13.56034	12.03237	5.22784
C	12.23117	13.45369	6.11529
H	11.95652	13.90642	5.16774
C	13.93858	9.44865	6.60531
H	13.00485	9.11067	7.06026
H	14.65348	8.62014	6.63993
H	13.74319	9.67872	5.55265
C	11.39322	13.88762	9.79338
H	11.72375	13.23315	10.60364
C	18.16666	15.13730	11.56685
H	18.86363	14.30398	11.68850
H	17.75013	15.38119	12.54772
H	18.73901	16.00381	11.22171
C	16.77798	9.68930	13.29935
H	16.27490	9.12867	12.50934
H	16.93207	9.02018	14.15118
H	17.75229	10.01428	12.92209
C	9.03795	10.99088	12.48922
H	9.85565	11.25705	11.81233
C	12.57618	9.38262	17.32343
C	15.84269	11.08791	6.77247
H	15.75660	11.37933	5.72046
H	16.55939	10.26260	6.83019
H	16.25663	11.93411	7.32543
C	7.88381	8.76156	12.76744
H	6.93503	9.28659	12.76547
C	7.73998	11.52787	11.89713
H	7.49353	11.03619	10.95257
H	7.83224	12.59983	11.70648
H	6.89441	11.39429	12.57957

C	13.70123	9.68685	18.31209
H	14.58545	9.07383	18.11778
H	13.36200	9.47245	19.33019
H	13.99845	10.73813	18.27506
C	11.37403	10.26281	17.69008
H	11.06764	10.08271	18.72653
H	10.51847	10.05966	17.04303
H	11.62565	11.32146	17.58451
C	12.18797	7.90840	17.49118
H	13.02448	7.25617	17.22601
H	11.34232	7.64101	16.85459
H	11.90649	7.70258	18.52976
C	16.17780	16.05089	10.41137
H	16.77527	16.92166	10.11985
H	15.67847	16.27765	11.35719
H	15.40518	15.90517	9.65409
C	11.64909	5.81697	11.32359
H	10.83560	5.08705	11.25539
H	12.59691	5.27550	11.23618
H	11.56581	6.49933	10.47441
C	9.32922	11.67087	13.82838
H	8.55177	11.42525	14.56000
H	9.35178	12.75931	13.71074
H	10.29103	11.35325	14.23284
C	11.78576	5.64892	13.83193
H	11.77112	6.20039	14.77425
H	12.74949	5.13590	13.75562
H	11.00682	4.88095	13.87473
C	11.88034	15.30295	10.10351
H	12.96748	15.33108	10.19820
H	11.44984	15.66070	11.04426
H	11.58820	16.00283	9.31330
C	9.86821	13.84395	9.74091
H	9.47365	14.49859	8.95729
H	9.44374	14.17661	10.69301
H	9.50794	12.83200	9.54303
C	17.74220	14.51926	9.20635
H	18.35182	15.37205	8.88766
H	17.00362	14.32134	8.42709
H	18.39017	13.64174	9.28133

Jones' one-coordinate amidogallium(I) system
75

Ga	-0.35602	2.35687	0.00416
N	-1.15737	0.55483	-0.00206
Si	-2.90053	0.38945	0.00488
C	-0.26166	-0.53473	-0.00639
C	-3.60009	2.08325	0.47379
C	-3.52013	-0.91001	1.23230
C	-3.66231	-0.08507	-1.66436
C	0.24112	-1.06613	-1.21412

C	0.23952	-1.06109	1.20559
H	-3.24770	2.41303	1.45507
H	-4.69196	2.02792	0.51570
H	-3.34779	2.85977	-0.25490
H	-2.99035	-1.85726	1.09682
H	-4.58392	-1.09916	1.06025
H	-3.40342	-0.59787	2.27272
H	-3.39877	0.62258	-2.45501
H	-4.75348	-0.09040	-1.57569
H	-3.35897	-1.08476	-1.98589
C	1.14401	-2.12263	-1.18956
C	-0.14989	-0.41409	-2.52929
C	1.14085	-2.11849	1.18710
C	-0.15241	-0.40321	2.51797
H	1.50343	-2.52325	-2.13227
C	1.59811	-2.67740	0.00012
H	-1.21193	-0.17121	-2.45333
C	-0.00380	-1.34004	-3.72527
C	0.55840	0.91395	-2.72958
H	1.49758	-2.51616	2.13208
H	-1.21467	-0.16023	2.43877
C	-0.00499	-1.32115	3.72018
C	0.55626	0.92539	2.71380
C	2.53415	-3.84996	0.00371
C	-0.92989	-2.37001	-3.89467
C	1.01991	-1.21521	-4.65903
C	-0.06934	1.92528	-3.45793
C	1.83312	1.15974	-2.22304
C	1.04797	-1.21877	4.62413
C	-0.95773	-2.31952	3.92615
C	1.83114	1.16907	2.20653
C	-0.07046	1.93857	3.44058
H	1.98079	-4.79480	0.02019
H	3.18355	-3.83950	0.88192
H	3.16525	-3.85907	-0.88764
H	-1.73142	-2.48649	-3.17197
C	-0.83548	-3.25006	-4.96023
C	1.11985	-2.09820	-5.72841
H	1.74615	-0.41668	-4.55581
H	-1.06173	1.74849	-3.86171
C	0.55482	3.14476	-3.67347
C	2.46481	2.37898	-2.44519
H	2.33390	0.38697	-1.65000
H	1.79459	-0.44338	4.49303
C	1.15142	-2.09443	5.69889
C	-0.85942	-3.19213	4.99791
H	-1.78533	-2.41418	3.23070
H	2.33067	0.39499	1.63415
C	2.46425	2.38794	2.42703
C	0.55502	3.15764	3.65413
H	-1.06358	1.76386	3.84361

H	-1.56906	-4.04120	-5.07256
C	0.19428	-3.11789	-5.88394
H	1.92605	-1.98219	-6.44489
H	0.04430	3.91942	-4.23529
C	1.82809	3.37558	-3.16813
H	3.45801	2.54955	-2.04393
H	1.98048	-1.99631	6.39156
C	0.19985	-3.08389	5.89049
H	-1.61403	-3.95849	5.13934
H	3.45749	2.55693	2.02524
C	1.82867	3.38617	3.14858
H	0.04524	3.93396	4.21433
H	0.27020	-3.80388	-6.72035
H	2.31611	4.32971	-3.33215
H	0.27852	-3.76406	6.73140
H	2.31767	4.34003	3.31116

(PON)Ga:
100

Ga	8.45787	5.49021	5.57706
P	7.04831	4.19844	3.17123
O	6.16750	4.37243	6.05970
N	8.60334	4.37286	7.21702
C	7.55279	3.83636	7.91351
C	5.13104	3.42066	8.01645
C	5.19196	3.81545	5.26104
C	9.87813	4.42196	7.84262
C	6.26318	3.85464	7.35303
C	7.02357	5.93957	2.54435
C	7.65679	3.26789	9.19301
H	8.63392	3.21626	9.65795
C	5.42343	3.70788	3.88669
C	5.99231	6.88792	2.68058
C	6.54050	2.78605	9.85479
H	6.65786	2.34896	10.84076
C	5.27901	2.86249	9.28538
H	4.41738	2.49786	9.82824
C	4.00258	3.38179	5.85207
C	10.83639	3.43057	7.55577
C	8.25835	6.34691	1.99345
C	4.44828	3.07529	3.11468
H	4.62307	2.93898	2.05449
C	7.13341	3.09226	1.68330
C	3.79488	3.65140	7.33237
C	11.48527	5.55241	9.24346
H	11.74667	6.37711	9.89885
C	7.40177	8.59354	1.64764
C	12.10182	3.52990	8.12743
H	12.84433	2.76836	7.91100
C	10.20563	5.49389	8.69942
C	9.20887	6.58208	9.04305

H	8.26230	6.32184	8.56444
C	8.41515	7.65277	1.54405
H	9.36912	7.94135	1.11173
C	6.20710	8.18551	2.22317
H	5.39910	8.90491	2.32326
C	12.43081	4.58107	8.96473
H	13.42289	4.64393	9.39897
C	10.51932	2.25516	6.65581
H	9.49433	2.39072	6.30355
C	9.42754	5.41481	1.86887
H	9.65080	4.93677	2.82994
H	10.31652	5.96061	1.55006
H	9.24374	4.61316	1.14868
C	3.27876	2.60555	3.68181
H	2.53560	2.11364	3.06457
C	6.65456	3.39415	0.39491
C	7.76276	1.85092	1.91162
C	3.05798	2.76742	5.04130
H	2.13464	2.40523	5.47535
C	4.64898	6.59827	3.28540
H	4.13299	5.77608	2.78707
H	4.01478	7.48344	3.21804
H	4.72766	6.32771	4.34040
C	8.95367	6.66444	10.54772
H	8.61345	5.70568	10.94424
H	8.18430	7.41157	10.76203
H	9.85692	6.95524	11.09177
C	9.65227	7.93833	8.49394
H	10.59700	8.25736	8.94356
H	8.90293	8.70456	8.71276
H	9.79492	7.90249	7.41081
C	5.90521	4.64921	0.04953
H	5.17912	4.91684	0.81782
H	5.37092	4.51471	-0.89274
H	6.57313	5.50669	-0.06159
C	7.60349	10.00885	1.19647
H	8.01830	10.61889	2.00544
H	6.66107	10.46812	0.89119
H	8.29898	10.06443	0.35651
C	3.40966	5.13516	7.50606
H	3.29359	5.36731	8.56760
H	2.46585	5.34440	6.99503
H	4.17642	5.79573	7.09706
C	7.93026	0.96236	0.85682
H	8.41177	0.00791	1.04992
C	6.85627	2.47149	-0.63074
H	6.48041	2.71136	-1.62161
C	2.68609	2.79252	7.92750
H	2.89916	1.72586	7.82862
H	1.72998	3.00574	7.44533
H	2.55913	3.02220	8.98654

C	7.49859	1.25886	-0.42962
C	8.23869	1.43766	3.27484
H	8.95938	2.14942	3.68375
H	8.71150	0.45510	3.23086
H	7.41195	1.37821	3.98893
C	11.43858	2.21848	5.43604
H	11.36740	3.14238	4.85557
H	11.17656	1.38264	4.78139
H	12.48501	2.09316	5.72793
C	10.57741	0.93283	7.42068
H	11.58612	0.73208	7.79380
H	10.29234	0.10131	6.76953
H	9.89795	0.94131	8.27568
C	7.72757	0.30389	-1.56227
H	8.72800	0.43704	-1.98649
H	7.00681	0.46045	-2.36706
H	7.65064	-0.73374	-1.23046

4. Crystallographic data

Table s1. Selected crystallographic and refinement data for compounds **2**–**7** and (PON)GaI_2.

	**2**	**3**·C_7H_8	**4**·C_6H_6	**5**·4/9(C_6H_6)	**6**·OEt_2	**7**·3/2(OEt_2)	(PON)GaI_2
Formula	$C_{45}H_{52}NOP$	$C_{52}H_{59}KNOP$	$C_{51}H_{57}GaNOP$	$C_{60}H_{66}GaNO_2P$	$C_{67}H_{76}BGaNO_3P$	$C_{69}H_{66}BF_{15}GaNO_{3.5}P$	$C_{50}H_{40}Ga_1O_1N_1P_1I_2$
Fw	653.89	784.07	800.66	3657.19	1054.78	1361.72	1050.47
Crystal System	Triclinic	Triclinic	Triclinic	Monoclinic	Monoclinic	Triclinic	Monoclinic
Space Group	P-1	P-1	P-1	$P2_1/n$	$P2_1/c$	P-1	$P2_1/n$
Wavelength/Å	1.54184	1.54184	1.54184	1.54184	1.54184	1.54184	1.54184
a/Å	11.3306(3)	12.7485(8)	12.8171(3)	12.6281(4)	21.9711(1)	12.9244(4)	11.7751(1)
b/Å	16.5217(6)	13.7483(9)	13.6400(4)	22.5013(5)	11.7926(1)	16.4616(5)	11.5701(1)
c/Å	20.7251(4)	14.3199(8)	14.2809(4)	18.1528(5)	23.0278(1)	17.5979(5)	34.8112(2)
α/°	81.490(2)	78.513(5)	78.240(2)	90	90	69.507(2)	90
β/°	84.932(2)	73.380(5)	72.984(2)	106.257(3)	106.760(1)	81.629(2)	95.360(1)
γ/°	79.593(2)	65.466(6)	65.459(3)	90	90	66.917(3)	90
Volume/$Å^3$	3766.34(19)	2178.5(3)	2161.97(13)	4951.9(2)	5712.97(7)	3226.18(18)	4721.91(6)
D_x/g cm^{-3}	1.153	1.195	1.230	1.226	1.226	1.402	1.478
Z	4	2	2	4	4	2	4
Temperature/K	150(2)	150(2)	150(2)	150(2)	150(2)	150(2)	150(2)
$R_1[I^2>2\sigma(I)]$	0.0499 (12588)	0.0562 (7332)	0.0469(8008)	0.0559 (7456)	0.0451 (10751)	0.0471 (10972)	0.0331 (9093)
wR_2[all data]	0.1416 (15583)	0.1594 (9036)	0.1554 (8973)	0.1547 (10337)	0.1329 (11922)	0.1415 (13577)	0.0877 (9825)
CCDC reference	2036920	2036921	2036922	2036923	2036924	2036925	2036919

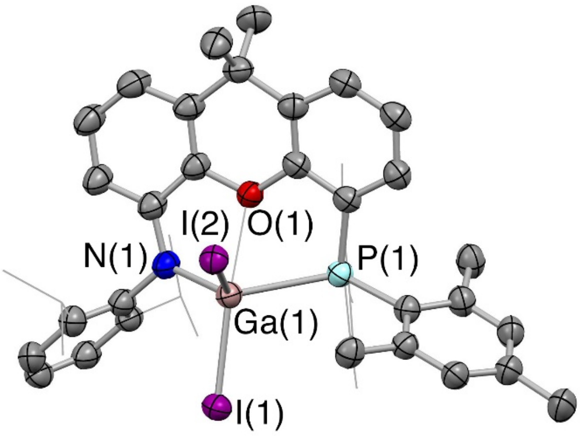

Figure s23. Molecular structure of (PON)GaI_2 in the solid state as determined by X-ray crystallography. Thermal ellipsoids set at the 40% probability level; H atoms and solvent molecules are omitted and selected groups shown in wireframe format for clarity. Key bond lengths (Å) and angles (°): Ga(1)-N(1) 1.927(3), Ga(1)-O(1) 2.388(2), Ga(1)-P(1) 2.612(1), Ga(1)-I(1) 2.570(1), Ga(1)-I(2) 2.580(1)
